# Sodium and Potassium Content of Foods Consumed in an Italian Population and the Impact of Adherence to a Mediterranean Diet on Their Intake

**DOI:** 10.3390/nu13082681

**Published:** 2021-08-01

**Authors:** Marcella Malavolti, Androniki Naska, Susan J. Fairweather-Tait, Carlotta Malagoli, Luciano Vescovi, Cristina Marchesi, Marco Vinceti, Tommaso Filippini

**Affiliations:** 1 Environmental, Genetic and Nutritional Epidemiology Research Center (CREAGEN), Department of Biomedical, Metabolic and Neural Sciences, University of Modena and Reggio Emilia, Via Campi 287, 41125 Modena, Italy; marcella.malavolti@unimore.it (M.M.); carlotta.malagoli@unimore.it (C.M.); vescovi.luciano@libero.it (L.V.); tommaso.filippini@unimore.it (T.F.); 2 Department of Hygiene, Epidemiology and Medical Statistics, School of Medicine, National and Kapodistrian University of Athens, Mikras Asias 75, 11527 Athens, Greece; anaska@med.uoa.gr; 3 Norwich Medical School, University of East Anglia, Norwich Research Park, James Watson Road, Norwich NR4 7UQ, UK; S.Fairweather-Tait@uea.ac.uk; 4 Head Office, Direzione Generale, Azienda USL-IRCCS di Reggio Emilia, Via Amendola 2, 42122 Reggio Emilia, Italy; Cristina.Marchesi@ausl.re.it; 5 Department of Epidemiology, Boston University School of Public Health, 715 Albany Street, Boston, MA 02118, USA

**Keywords:** dietary intake, Mediterranean diet, potassium, public health, salt, sodium, sodium-to-potassium ratio

## Abstract

High sodium and low potassium intakes are associated with increased levels of blood pressure and risk of cardiovascular diseases. Assessment of habitual dietary habits are helpful to evaluate their intake and adherence to healthy dietary recommendations. In this study, we determined sodium and potassium food-specific content and intake in a Northern Italy community, focusing on the role and contribution of adherence to Mediterranean diet patterns. We collected a total of 908 food samples and measured sodium and potassium content using inductively coupled plasma mass spectrometry. Using a validated semi-quantitative food frequency questionnaire, we assessed habitual dietary intake of 719 adult individuals of the Emilia-Romagna region. We then estimated sodium and potassium daily intake for each food based on their relative contribution to the overall diet, and their link to Mediterranean diet patterns. The estimated mean sodium intake was 2.15 g/day, while potassium mean intake was 3.37 g/day. The foods contributing most to sodium intake were cereals (33.2%), meat products (24.5%, especially processed meat), and dairy products (13.6%), and for potassium they were meat (17.1%, especially red and white meat), fresh fruits (15.7%), and vegetables (15.1%). Adherence to a Mediterranean diet had little influence on sodium intake, whereas potassium intake was greatly increased in subjects with higher scores, resulting in a lower sodium/potassium ratio. Although we may have underestimated dietary sodium intake by not including discretionary salt use and there may be some degree of exposure misclassification as a result of changes in food sodium content and dietary habits over time, our study provides an overview of the contribution of a wide range of foods to the sodium and potassium intake in a Northern Italy community and of the impact of a Mediterranean diet on intake. The mean sodium intake was above the dietary recommendations for adults of 1.5–2 g/day, whilst potassium intake was only slightly lower than the recommended 3.5 g/day. Our findings suggest that higher adherence to Mediterranean diet patterns has limited effect on restricting sodium intake, but may facilitate a higher potassium intake, thereby aiding the achievement of healthy dietary recommendations.

## 1. Introduction

Non-communicable diseases (NCDs) are the leading causes of death worldwide [1]. In particular, cardiovascular diseases (CVD) still represent the leading cause of death and disability [2], being responsible for more than 35% of all deaths in Italy [3]. Although effective preventive measures can be implemented; i.e., by controlling behavioral and metabolic factors, nutritional determinants represent the most significant risk factors, preceded exclusively by blood pressure [4,5,6,7].

The effect of dietary intakes of sodium and potassium on the regulation of blood pressure levels has been widely investigated [8]. Sodium and potassium have a fundamental role in the distribution of fluids inside and outside cells, and in osmolarity regulation. Differences in sodium and potassium concentrations are maintained by the specific permeability of cell membranes and by activity of transmembrane transporters such as Na/K-ATPase. In particular, potassium is an essential mineral that plays a primary role in several physiological mechanisms including transmission of electrical activity in muscle cells and nerve fibers [1,9]. In humans, an increase in potassium intake has been linked to decreasing blood pressure levels and stroke incidence, with an indication of a possible U-shape relationship with minimum risk at 3.5 g/day (i.e., 90 mmol/day) of potassium excretion [10,11]. Conversely, a decrease in sodium intake showed an approximately linear association with blood pressure levels [12], cardiovascular diseases [13], and stroke [14], with beneficial effects of sodium reduction in both subjects with and without hypertension [12,15,16]. 

In addition to CVD, blood pressure lowering has also been associated with a reduced risk of mild cognitive impairment [17]. There is growing evidence that a high salt intake is detrimental for cognition [18,19], thus a reduction in sodium together with an increase in potassium intake, plus other dietary interventions, may be helpful for dementia prevention [19,20,21]. For these reasons, the World Health Organization (WHO) has approved an action plan for reducing the costs of public health, one of which is to lower salt consumption by the population [22].

In Italy, few studies have estimated dietary sodium and potassium intakes at the population level. Findings from the Italian Total Diet Study 2001–2002 using a 7 day diary recall reported an average intake of 3.8 and 2.9 g/day for sodium and potassium, respectively [23]. More recently, a lower sodium intake has been reported, namely 2.3 and 1.9 g/day for men and women, respectively [24]. Nonetheless, these values are still higher than the recommended dietary intake (DRI) of 1.5 and 2.0 g/day, respectively, suggested by the Italian Society of Human Nutrition (SINU) [25] and the safe and adequate intake of 2.0 g/day for adults suggested by the European Food Safety Authority (EFSA) [26]. As regards potassium, a slight increase in its intake was seen in the past years, from 3.2 g/day and 2.8 g/day in men and women, respectively, during the Third Italian National Consumption Survey in 2005–2006 using a 3-day semi-structured diary [27], to 3.3 g/day and 3.0 g/day in men and women, respectively, during the Cardiovascular Epidemiology Observatory/Health Examination Survey (CUORE) in 2008–2012 using 24 h urine collection carried out by the Italian National Institute of Health [24]. However, the latest 2018–2019 data from the CUORE project conducted by the Italian National Institute of Health highlighted that the potassium intake of the Italian general adult population has fallen to 3.043 g/day for men and 2.561 g/day for women [28]. This highlights the importance of increasing potassium intake in order to meet the adequate intakes of 3.5 g/day or 3.9 g/day suggested by the EFSA and SINU, respectively [9,25]. 

The primary aim of the present study was to assess dietary intakes of sodium and potassium in a sample population from the Emilia-Romagna region in Northern Italy, and to evaluate how such intakes may be influenced by adherence to dietary patterns widely known to be beneficial to human health, namely those related to the Mediterranean diet.

## 2. Materials and Methods

### 2.1. Food Collection and Analysis

We measured the sodium and potassium content in food samples collected from markets and stores in Northern Italy, as previously described [29,30]. Briefly, we identified foods most frequently consumed in a typical Italian diet [31,32]. We selected relevant food items characterizing the Italian diet [33], with particular reference to the Emilia-Romagna Region using previous population-based studies addressing the dietary habits of subjects of this Northern Italy community [34,35]. 

We purchased selected food and beverages in two provinces of the Emilia-Romagna region, namely Modena and Reggio Emilia in the period of October 2016–February 2017. During sample handling, we tried to avoid metal cross-contamination using plastic food containers and plastic and stainless cutlery. We also used a food blender equipped with a stainless-steel blade. After homogenisation, we aliquoted 0.5 g portion (wet weight) into quartz containers previously washed with MilliQ water (MilliQPlus, Millipore, MA, USA) and HNO_3_. Sample digestion was performed using 10 mL solution (5 mL HNO_3_ + 5 mL H_2_O) in a microwave system (Discover SP-D, CEM Corporation, NC, USA). Digested material was stored in plastic tubes and diluted to 50 mL with deionised water before analysis. We measured the sodium and potassium concentration using an inductively coupled plasma-mass spectrometer (Agilent 7500ce, Agilent Technologies, Santa Clara, CA, USA). All analyses were performed in duplicate, implementing standard quality controls as previously described [36,37]. A total of 908 food study samples were analyzed. Limits of quantification were 0.02 and 0.10 mg/kg for sodium and potassium, respectively, with corresponding limits of detection (LOD) of 0.01 and 0.05 mg/kg. Values for 35 and 9 samples for sodium and potassium, respectively, were below the LOD and were set equal to LOD/2 [38]. 

### 2.2. Study Population and Assessment of Dietary Habits

We assessed habitual dietary intakes of a Northern Italy community in the period 2005–2006. We identified a large population sample from the databases of the Emilia-Romagna Region–National Health Service directories of five provinces (Bologna, Ferrara, Modena, Parma and Reggio Emilia). Inclusion criteria were age > 18 years, and residence in one of the studied provinces. From the random sample of 2825 eligible subjects invited to participate, a total of 747 subjects accepted (response rate of 26.4%), and were therefore recruited. The study was authorized by the Modena Ethics Committee (approval no. 71.11/2011). All participants provided written informed consent and returned study material for collection of individual characteristics.

To estimate dietary habits, we administrated the food frequency questionnaires (FFQ) implemented within the ‘European Prospective Investigation into Cancer and Nutrition’ project. The EPIC-FFQ is a validated semi-quantitative FFQ, and we used the version specifically developed for the population of Northern Italy [39]. Frequency and amount of consumption of 188 food items were estimated over the previous year. Accurate completion by participants was ensured through photos of foods and serving sizes. For quality data control, we excluded incomplete FFQs or those reporting extreme and implausible values of energy intake; i.e., <0.5th or >99.5th percentile, based on the ratio of total energy intake to calculated basal metabolic rate, which excluded 28 subjects [40]. The final study sample was 719 adult participants, including 319 men and 400 women, none of whom reported ‘special’ dietary requirements or habits such as weight control or vegetarian/vegan diet during the survey. We eventually estimated daily dietary intake (mg/day) as previously reported by multiplying the quantity of sodium and potassium in foods (mg/kg food as consumed) with the corresponding food intake determined from the FFQ (in g/day) [41] and we calculated the sodium-to-potassium (Na/K) ratio. For daily dietary intakes, we also performed stratified analysis by sex and by adherence to the Italian Mediterranean Index (IMI) [42], and to the Mediterranean–DASH Intervention for Neurodegenerative Delay (MIND) pattern, which combines the Dietary Approach to Stop Hypertension (DASH) and the Mediterranean diet, with the aim of reducing the risk of dementia [43,44]. We used frequency and quantity of food and beverage intake as assessed through the EPIC-FFQ to evaluate the adherence to the investigated dietary patterns. In particular, we implemented specifically developed routines for the scoring system of both the IMI and MIND diets.

The Italian Mediterranean Index (IMI) was developed by the Epidemiology and Prevention Unit of the Milan National Cancer Institute [45] by adapting the Greek Mediterranean Index to typical Italian eating behavior. The score is based on intake of 11 items: 6 typical Mediterranean foods or food groups (pasta; typical Mediterranean vegetables such as raw tomatoes, leafy vegetables, onion and garlic, salad, and fruiting vegetables; fruit; legumes; olive oil; and fish), 4 non-Mediterranean foods (soft drinks, butter, red meat, and potatoes), and alcohol consumption. One point was given for consumption of each typical Mediterranean food in the upper tertile of the distribution, and for consumption of each non-Mediterranean food in the bottom tertile; all other dietary components received 0 points. Alcohol received 1 point for intake up to 12 g/day; abstainers and persons who consumed >12 g/day did not receive any points. The range of possible scores was 0–11. Similarly, the MIND pattern has 15 components, including 10 brain-healthy food groups (green leafy vegetables, other vegetables, nuts, berries, beans, whole grains, white meat (fish and poultry), olive oil instead of other oil, and wine-intake limitation), as well as a decrease in 5 unhealthy food groups, including red meats, butter and stick margarine, cheese, pastries and sweets, and fried/fast food. Scores range from 0 to 15, with higher values meaning greater adherence [46]. Additional general guidelines for the MIND diet are consuming at least three servings of whole grains, a salad and one other vegetable, and a glass of wine each day. In addition, nuts are used as a snack on most days and beans every other day. Poultry and berries are recommended at least twice a week and fish at least once a week. It is essential to limit the intake of the MIND diet’s “unhealthy food groups”, especially butter (less than 1 tablespoon a day), cheese, and fried or fast food (less than a serving a week for any of the three) [46]. For both diets, we used the mean value as cutpoint for identification of poor and high adherence.

We checked the normal distribution of variables using the Shapiro–Wilk test, and we used a *t*-test for independent samples to compare differences between means. We used linear regression analysis to investigate the relation between the Na/K ratio and adherence score to both IMI and MIND patterns, adjusting for age, sex, body mass index, and energy intake. We reported beta regression coefficients and values of the predicted Na/K ratio together with their 95% confidence intervals (CI). We used Stata statistical package (v17.1 Stata Corp., College Station, TX, USA, 2021) for all statistical analyses.

## 3. Results

Characteristics of the study participants are reported in Table 1. Out of the total 719 included participants, there were 319 men, median age 60 (interquartile range-IQR: 50–69) years and 400 women, median age 53 (IQR: 41–63) years. Nearly half of the participants had educational attainment of high school or more, and the vast majority lived with partners (married or unmarried). Body mass index distribution showed a general tendency to overweight with a median value of 25.1. The median energy intake was 1907 kcal/day, higher in men (2025 kcal/day) than in women (1801 kcal/day). Adherence to the Italian Mediterranean Index was moderate with approximately 60% of subjects having a score equal or above the median value of 4, and a similar higher proportion of subjects appeared to adhere to MIND pattern, with a mean score of 7.5. Mean intake of specific food categories demonstrated comparable dietary habits in both sexes, except for a slightly lower consumption of cereal products (206.4 g/day vs. 174.2 g/day), meat (142.4 g/day vs. 117.2 g/day) and beverages (mainly due to wine: 191.4 g/day vs. 73.9 g/day) in women, while men had a lower consumption of milk and yogurt (159.4 g/day vs. 212.5 g/day), coffee and tea (120.6 g/day vs. 170.6 g/day) and a slightly lower intake of vegetables (154.1 g/day vs. 161.4 g/day) and citrus fruits (207.0 g/day vs. 223.8 g/day) (Supplementary Table S1).

The average sodium and potassium content of food is reported in Table 2. The highest sodium levels were found in processed meat (12.0 g/kg); fish and seafood, especially preserved and tinned fish (13.0 g/kg); followed by bread, rolls, and other prepackaged cereals and salty snacks (5.4 and 6.2 g/kg, respectively). In addition, cheese (5.2 g/kg), butter, and other animal fats (3.7 g/kg) showed a higher sodium content compared to milk (4.4 g/day) and vegetable/olive oils (<0.3 g/kg), respectively. Conversely, the highest potassium levels were found in legumes (10.0 g/kg); dry fruits (7.2 g/kg); followed by meat, especially white meat (5.3 g/kg); mushrooms (5.1 g/kg); vegetables (3.1 g/kg); and potatoes (4.1 g/kg). Food containing lower sodium levels were fresh fruits (14.8 mg/kg), followed by almost all beverages (35.4 mg/kg), while rice demonstrated the lowest value (8.0 mg/kg) compared to other cereal products. Lower levels of potassium were found in almost all dairy products (1.2 g/kg), beverages (0.5 g/kg), and oils and fats (0.1 g/kg).

The dietary intakes of sodium and potassium in the study population are reported in Table 3 and Table 4. Overall mean intakes were 2.2 g/day and 3.4 g/day for sodium and potassium, respectively. Foods contributing the most to sodium intake were cereals (33.2%); meat products (24.5%), mainly processed meats; followed by dairy products (13.6%) and vegetables (12.6%). For potassium, they were meat (17.1%), fresh fruits (15.7%), and vegetables (15.1%), and with relevant contributions from cereals and dairy products (11.6% each) and beverages (10.8%). Men had overall intakes that were only slightly higher than women for both sodium (2.2 vs. 2.1 g/day) (Supplementary Tables S2 and S3) and potassium (3.4 vs. 3.3 g/day) (Supplementary Tables S4 and S5).

**Figure 1:**
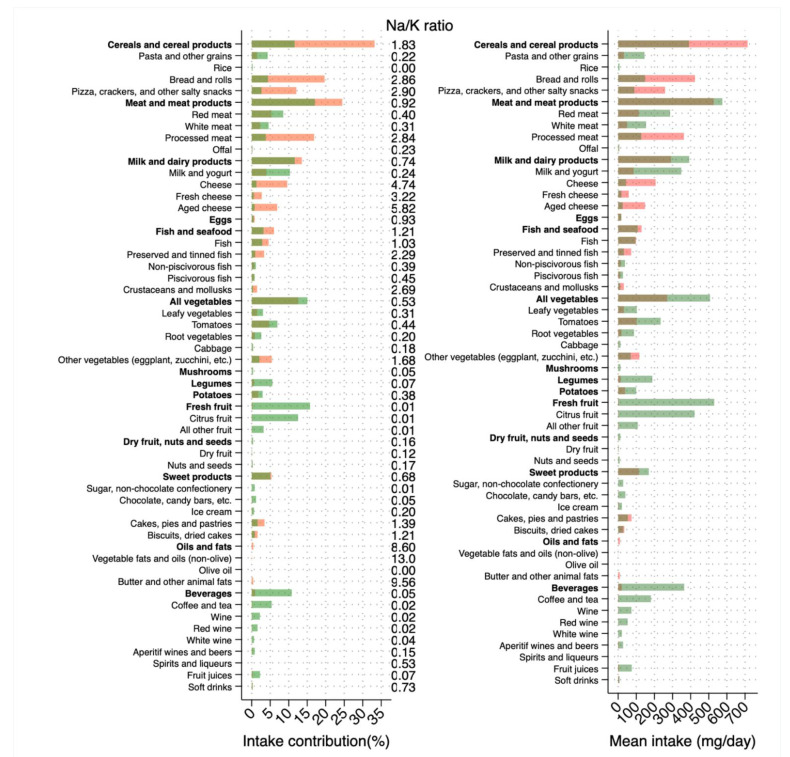
Average level of intake (in mg/day) of sodium and potassium according to food categories, their percentage contribution (in %) and sodium-to-potassium (Na/K) ratio. Light and dark orange bars indicate sodium and light and dark green bars indicate potassium, respectively.

In Figure 1, we present a comparison between sodium and potassium intake from all food categories, their contribution to the overall intake, and the food-specific Na/K ratio. Cereals overall had a higher Na/K ratio, although pasta and rice had a lower Na/K ratio. Similarly, fish and seafood favored sodium with high Na/K ratios for preserved/tinned fish and crustaceans/mollusks, while other types of fish showed the opposite relationship. Similar patterns were noted for dairy products with Na/K ratio in favor of potassium for milk and yogurt, but not for cheese. Interestingly, legumes, potatoes and particularly fresh fruits intakes were in favor of potassium and the same for beverages, mainly driven by coffee, tea and wine. It is noteworthy that amongst oils and fats, olive oil only showed a Na/K ratio in favor of potassium whereas the intake of both animal fats and other vegetable oils greatly increased sodium intake.

**Figure 2:**
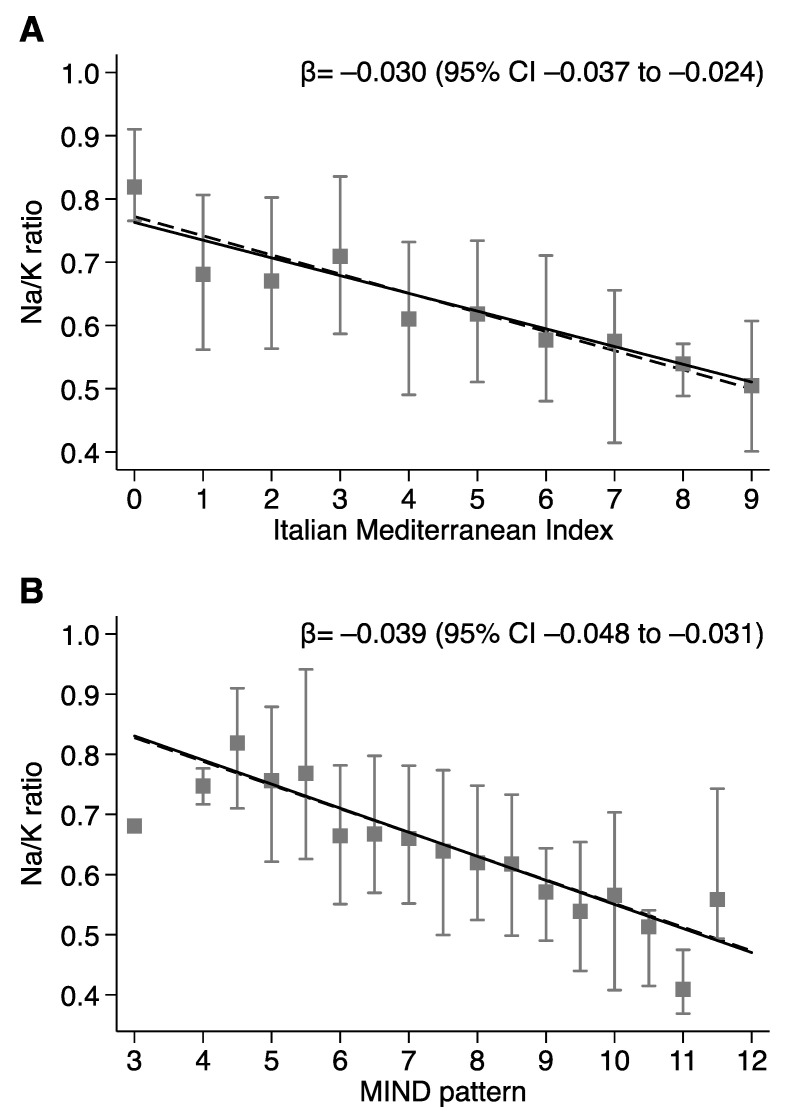
Linear regression between sodium-to-potassium (Na/K) ratio and Italian Mediterranean Index-IMI (**A**) or MIND pattern (**B**). Squares and spikes indicate median and interquartile range, respectively. Solid and dashed lines indicate crude and adjusted linear fitting, respectively.

The distribution of sodium and potassium intake divided below (<4) and above (≥4) the median adherence to the Italian Mediterranean Index showed no substantial influence on sodium intake (2.2 vs. 2.1 g/day for low and high adherence, respectively) (Table 5). Conversely, subjects with adherence above the median score of the Italian Mediterranean Index demonstrated substantial higher potassium intake (3.1 vs. 3.6 g/day). Substantially identical findings were observed when dividing below (<7.5) and above (≥7.5) the median adherence to the MIND pattern (Table 6). Finally, the increase in adherence to Mediterranean and MIND patterns was linearly associated with a decrease in the Na/K ratio, from a predicted value based on linear regression analysis of 0.77 (95% CI 0.74–0.80) in the subjects with lowest adherence to the predicted value of 0.50 (95% CI 0.47–0.53) in the highest-adherent ones (Figure 2). Similar relationships were noted with the MIND pattern, with a predicted Na/K ratio of 0.83 (95% CI 0.79–0.87) in the lowest-adherence subjects and 0.47 (95% CI 0.43–0.51) in the highest ones (Figure 2).

## 4. Discussion

In this Northern Italy community, we found that the mean sodium intake was above the current internationally recognized dietary recommendations of 1.5–2 g/day for adult populations [26], while the mean potassium intake was slightly lower than the dietary recommendation of 3.5 g/day [9]. Compared to previous Italian surveys, we found a small decrease in the average sodium intake when compared to the 3.8 g/day reported by the Italian Total Diet Study (TDS) 2001–2002 [23]. The most recent 2008–2012 CUORE survey showed substantially similar intakes with values of 2.3 and 1.9 g/day for men and women, respectively [24]. As regards potassium, our results were similar to intakes reported in the recent surveys which generally reported a potassium intake >3 g/day [24,27,47], although differences between sexes were less marked compared to other surveys, without an appreciably higher intake in men compared to women [27,28]. As a consequence, it is not surprising that higher adherence to the Mediterranean and MIND diets is associated with a general decrease of Na/K ratio. That lowering effect seemed mainly driven by an increase in potassium intake, since sodium intake was only slightly affected while potassium increased at higher levels of adherence to the investigated dietary patterns.

Despite the fact that there is no single definition of Mediterranean diet, it usually describes the traditional diet of populations residing in areas bordering the Mediterranean Sea, and is characterized by a high consumption of plant-based foods (such as vegetable, legumes, fruits, and nuts), preference for whole-grain cereals, fish and dairy products instead of other sources of refined carbohydrates and animal proteins [48,49]. Other features include the daily consumption of olive oil and moderate intake of alcohol (mostly red wine) during meals [49]. However in recent decades, there has been a gradual decline in adherence to the traditional Mediterranean diet in favor of Western dietary habits characterized by an unbalanced intake of foods that may lead to micronutrient deficiencies [50].

In spite of the general assumption of high adherence of Italians to a Mediterranean dietary pattern, this is unfortunately not true, since a gap still exists between the recommendations and the real dietary pattern, especially in most recent years [51,52]. Habitual salt intake in Europe is approximately 10 g per day [53] of which unprocessed foods represented approximately 12% of total salt intake but the majority (over 75%) occurred from processed food consumption [53,54,55] rather than from discretionary salt. The assessment of dietary sodium intake in free-living individuals is challenging due to high day-to-day variation, diversity in sources (naturally present, added during food processing, and discretionary use of salt at home) and the general trend for an overall reduction in salt concentrations of processed foods in recent years.

Our findings suggest that increased adherence to IMI and MIND patterns has limited effects on sodium intake, but it leads to an almost adequate potassium intake. For individuals, WHO recommend a sodium intake <2 g/day and a potassium intake >3.5 g/day [56,57] which would yield a Na/K ≤0.6, a ratio of intake considered beneficial for health in order to decrease blood pressure and generally improve cardiovascular health [52,58]. Foods showing the lowest Na/K ratio were fresh and dry fruits, most vegetables, legumes, and beverages, in accordance with previous surveys [47,59]. Conversely, among other categories, processed and prepackaged cereals (particularly salty and sweet snacks), processed meat, cheese, and non-olive oils and fats demonstrated higher values according to our findings and those reported previously [59,60]. In addition, efforts to decrease Na/K ratio could include specific indications supporting the use of low-sodium products, the gradual reduction of added salt to allow progressive adaptation towards a more sensitive taste, and the use of salt substitutes rich in potassium [61,62].

Our study population is generally characterized by good adherence to the Mediterranean diet [63]. However, more than a third of subjects (38%) in the study population had scores between 0–3 out of a maximum score of 11 points, indicating that improvement in dietary habits is both possible and definitely needed. Various longitudinal studies have analyzed the benefits of a Mediterranean diet in comparison with other types of diet [64,65,66,67]. These studies have shown that people with good adherence to a Mediterranean diet have a better quality of life and greater life expectancy, along with a decreased prevalence of chronic diseases [68,69]. Evidence has been accumulating that the Mediterranean diet may provide a substantial benefit by reducing the risk of many chronic diseases such as CVD disease, e.g., through maintenance of blood pressure and endothelial function [70,71].

Our study has some limitations. We did not include the quantity of drinking water in the intake of beverages, but it is considered a trivial source of both sodium and potassium [55,72,73]. In addition, we did not account for discretionary salt use since the EPIC questionnaire only assessed the habit to add salt when subjects do not eat at home. Moreover, we recruited only healthy adult subjects, and the results cannot be generalized to other populations e.g., children and the elderly, the latter being potentially more vulnerable to adverse effects in relation to excess sodium intake. In addition, since the recruitment period was carried out during 2005–2006, dietary habits of the population may have changed over time, although recent results from 2008–2012 showed similar values of intake of sodium and potassium [24], suggesting a relatively stable intake in a population without intervention specifically aimed at improving dietary habits. Finally, considering the ten-year gap between the time of population sampling and food analysis, we cannot entirely rule out that some changes over time may have occurred towards a sodium reduction and thus partially biased our estimates, especially due to a decrease in salt in processed foods [74]. Small, though still limited, changes in the sodium content of processed and restaurant foods appear to have occurred in more recent years [75,76,77]. However, the relatively low intake of processed and prepackaged foods in the study population, and the evidence that high sodium levels are still found in these products, despite efforts by the food industry in lowering its concentrations [78] suggests that our assessments are likely still currently valid.

One strength of our study is that we measured the sodium and potassium content of a large number of food samples and beverages that represent habitual dietary patterns of Northern Italy, and were therefore able to assess sodium and potassium dietary intakes with a high degree of accuracy. In addition, we estimated dietary intakes in a randomly selected large population sample from the Emilia-Romagna region. Although its dietary characteristics could be generally similar to those of other Italian populations [45,79], some peculiarities may be present due to the regional differences in plant growing and animal husbandry. However, the 2008–2012 survey was carried out with recruitment of participants from 21 municipalities across the country [24], among which Modena municipality only has been considered, and in addition no specific data about sodium and potassium was made available [80].

Finally, as regards the assessment of dietary habits, we estimated both sodium and potassium intake along with adherence to Mediterranean diet in our study community through implementation of the EPIC-validated semi-quantitative FFQ in the version tailored for dietary habits of the population of Northern Italy [81]. Although there is a European consensus that two non-consecutive 24 h dietary recalls are the preferred methods for the estimation of sodium intake when using a dietary survey at the population or group level [82], the implementation of the EPIC-FFQ should be considered a strength of the study. The EPIC-FFQ assesses frequency and quantity of consumption over an entire year, and takes into account intakes of seasonal food, supporting its substantial validity and reproducibility for the assessment of dietary habits in the adult population [83].

## 5. Conclusions

Our study provides an updated assessment of foods that contribute to sodium and potassium intake in a Northern Italy community. We found a mean sodium intake of 2.2 g/day, which exceeds the dietary recommendation for adults of 1.5–2 g/day, whereas the mean potassium intake was 3.4 g/day, which was slightly lower than the recommended 3.5 g/day. Our findings suggest that higher adherence to Mediterranean diet and MIND patterns has no effect on sodium intake, but may have a beneficial influence on potassium, favoring the achievement of the recommended dietary intake though a decrease in the Na/K ratio, notably by higher intakes of fresh and dry fruits, most vegetables, legumes, and beverages, as well as a reduction in processed and prepackaged foods.

## Figures and Tables

**Table 1:** Characteristics of study participants. Number (percentage-%) reported when not otherwise indicated and median and interquartile range (IQR).

	All Subjects		Men		Women	
Characteristics	N	(%)	N	(%)	N	(%)
Overall	719	(100)	319	(44.4)	400	(55.6)
Age (years)						
Median (IQR)	57	(43–67)	60	(50–69)	53	(41–63)
<65 years	499	(69.4)	190	(59.6)	309	(77.2)
≥65 years	220	(30.6)	129	(40.4)	91	(22.8)
Education (years)						
≤5, primary school or less	170	(23.6)	86	(27.0)	84	(21.0)
6–8, middle school	178	(24.8)	86	(27.0)	92	(23.0)
9–13, high school	268	(37.3)	101	(31.7)	167	(41.7)
≥14, college or more	103	(14.3)	46	(14.4)	57	(14.3)
Marital status						
Married/unmarried partner	493	(68.6)	239	(74.9)	254	(63.5)
Unmarried/single	104	(14.5)	42	(13.2)	62	(15.5)
Divorced	48	(6.7)	18	(5.6)	40	(7.5)
Widowed	74	(10.3)	20	(6.3)	54	(13.5)
Body mass index (kg/m^2^)						
Median (IQR)	25.1	(22.7–27.8)	26.1	(24.2–28.7)	24.1	(21.6–27.0)
≤19	45	(6.3)	3	(0.9)	42	(10.5)
20–24	306	(42.6)	116	(36.4)	190	(47.5)
25–29	287	(39.9)	162	(50.8)	125	(31.2)
≥30	81	(11.3)	38	(11.9)	43	(10.8)
Energy (kcal/day) Median (IQR)	1907	(1538–2364)	2025	(1650–2463)	1801	(1456–2296)
Italian Mediterranean Index						
Median (IQR)	4	(3–5)	4	(3–5)	4	(3–5)
<4	275	(38.2)	129	(40.4)	146	(36.5)
≥4	444	(61.8)	190	(59.6)	254	(63.5)
MIND pattern						
Median (IQR)	7.5	(6.5–8.5)	7.5	(6.5–8.5)	7.5	(7.0–8.5)
<7.5	288	(40.1)	144	(45.1)	144	(36.0)
≥7.5	431	(59.9)	175	(54.9)	256	(64.0)

**Table 2:** Average content (mg/kg wet weight) of sodium (Na) and potassium (K) in foods and number (%) of samples below the limit of detection (LOD).

	Sodium				Potassium			
Food ^1^	N	Mean Na	<LOD (N)	<LOD (%)	Mean K	<LOD (N)	<LOD (%)	Na/K Ratio
**Cereals and cereal products**	**112**	**3397.4**	**2**	**1.8**	**2094.8**	**0**	**0.0**	**1.62**
Pasta and other grains	41	551.4	1	2.4	2544.2	0	0.0	0.22
Rice	8	8.0	1	12.5	1443.9	0	0.0	0.01
Bread and rolls	42	5409.8	0	0.0	1888.7	0	0.0	2.86
Pizza, crackers, and other salty snacks	21	6220.2	0	0.0	1877.3	0	0.0	3.31
**Meat and meat products**	**86**	**6037.9**	**0**	**0.0**	**4275.9**	**0**	**0.0**	**1.41**
Red meat	28	1715.9	0	0.0	4020.9	0	0.0	0.43
White meat	12	1637.6	0	0.0	5258.2	0	0.0	0.31
Processed meat	36	12,000.0	0	0.0	4329.0	0	0.0	2.77
Offal	10	843.4	0	0.0	3620.1	0	0.0	0.23
**Milk and dairy products**	**72**	**4356.2**	**0**	**0.0**	**1178.1**	**0**	**0.0**	**3.70**
Milk and yogurt	13	416.5	0	0.0	1726.4	0	0.0	0.24
Cheese	59	5224.2	0	0.0	1057.3	0	0.0	4.94
Fresh cheese	17	4002.5	0	0.0	1241.9	0	0.0	3.22
Aged cheese	42	5718.8	0	0.0	982.6	0	0.0	5.82
**Eggs**	**9**	**1110.8**	**0**	**0.0**	**1192.7**	**0**	**0.0**	**0.93**
**Fish and seafood**	**62**	**4271.7**	**0**	**0.0**	**2789.1**	**0**	**0.0**	**1.53**
Fish	41	4251.5	0	0.0	3407.6	0	0.0	1.25
Preserved and tinned fish	9	13,000.0	0	0.0	3572.0	0	0.0	3.64
Non-piscivorous fish	15	1430.8	0	0.0	3541.6	0	0.0	0.40
Piscivorous fish	17	2079.5	0	0.0	3202.4	0	0.0	0.65
Crustaceans and mollusks	21	4311.3	0	0.0	1581.5	0	0.0	2.73
**All vegetables**	**196**	**1846.7**	**2**	**1.0**	**3056.9**	**0**	**0.0**	**0.60**
Leafy vegetables	40	908.9	0	0.0	3307.6	0	0.0	0.27
Tomatoes	19	2285.4	0	0.0	4244.1	0	0.0	0.54
Root vegetables	14	435.6	1	7.1	2662.1	0	0.0	0.16
Cabbage	28	579.2	0	0.0	3140.3	0	0.0	0.18
Other vegetables	63	3903.5	1	1.6	2790.8	0	0.0	1.40
**Mushrooms**	**5**	**218.2**	**0**	**0.0**	**5093.9**	**0**	**0.0**	**0.04**
**Legumes**	**43**	**725.9**	**11**	**25.6**	**10,062.5**	**0**	**0.0**	**0.07**
**Potatoes**	**14**	**1543.8**	**0**	**0.0**	**4054.7**	**0**	**0.0**	**0.38**
**Fresh fruit**	**60**	**14.8**	**3**	**5.0**	**1902.6**	**0**	**0.0**	**0.01**
Citrus fruit	12	24.7	1	8.3	1712.9	0	0.0	0.01
All other fruit	48	12.3	2	4.2	1950.0	0	0.0	0.01
**Dry fruit, nuts and seeds**	**45**	**1138.4**	**7**	**15.6**	**7189.2**	**0**	**0.0**	**0.16**
Dry fruit	8	1149.8	0	0.0	8351.2	0	0.0	0.14
Nuts and seeds	37	1135.9	7	18.9	6938.0	0	0.0	0.16
**Sweets products**	**79**	**1408.0**	**1**	**1.3**	**3271.8**	**0**	**0.0**	**0.43**
Sugar, non-chocolate confectionery	8	128.6	0	0.0	2078.2	0	0.0	0.06
Chocolate, candy bars, etc.	21	392.2	1	4.8	7529.2	0	0.0	0.05
Ice-cream	5	297.0	0	0.0	1490.8	0	0.0	0.20
Cakes, pies and pastries	30	2108.8	0	0.0	1515.8	0	0.0	1.39
Biscuits, dry cakes	15	2481.4	0	0.0	2053.5	0	0.0	1.21
**Oils and fats**	**23**	**1305.4**	**8**	**34.8**	**121.8**	**0**	**0.0**	**10.72**
Vegetables fats and oils (non-olive)	12	332.2	5	41.7	24.2	7	58.3	13.73
Olive oil	4	1.3	3	75.0	6.1	1	25.0	0.21
Butter and other animal fats	7	3718.9	0	0.0	355.3	0	0.0	10.47
**Beverages**	**102**	**35.4**	**1**	**0.9**	**534.2**	**1**	**0.9**	**0.07**
Coffee and tea	8	21.4	0	0.0	1236.5	0	0.0	0.02
Wine	50	14.7	0	0.0	566.1	0	0.0	0.03
Red wine	27	14.3	0	0.0	708.2	0	0.0	0.02
White wine	23	15.1	0	0.0	399.3	0	0.0	0.04
Aperitif wines and beers	8	53.3	1	12.5	493.3	0	0.0	0.11
Spirits and liqueurs	21	45.8	0	0.0	60.9	1	4.8	0.75
Fruit juices	8	52.2	0	0.0	1229.5	0	0.0	0.04
Soft drinks	7	128.6	0	0.0	175.5	0	0.0	0.73

^1^ Bold characters relate to main food categories.

**Table 3:** Distribution of sodium daily dietary intake in all subjects (in mg/day) and percentage contribution (%) of each food to total intake.

Food ^1^	Mean	SD	P5	P25	P50	P75	P95	%
**Total intake**	**2151.8**	**867.9**	**1019.9**	**1563.1**	**2025.9**	**2569.0**	**3785.1**	**100**
**Cereals and cereal products**	**714.3**	**453.5**	**110.2**	**396.7**	**661.7**	**943.2**	**1487.9**	**33.20**
Pasta and other grains	31.5	22.4	2.3	15.9	27.6	42.8	74.5	1.46
Rice	0.0	0.1	0.0	0.0	0.0	0.0	0.2	0.00
Bread and rolls	423.8	389.3	0.0	107.7	324.6	587.5	1112.8	19.70
Pizza, crackers, and other salty snacks	259.0	191.4	31.2	128.6	219.1	354.0	581.7	12.04
**Meat and meat products**	**526.5**	**349.8**	**125.6**	**290.9**	**455.1**	**670.1**	**1222.3**	**24.47**
Red meat	113.9	79.0	14.0	57.6	100.0	153.5	267.7	5.29
White meat	48.1	42.7	0.7	18.9	38.8	67.0	131.6	2.24
Processed meat	363.0	306.5	32.0	151.4	284.3	478.8	957.7	16.87
Offal	1.5	3.9	0.0	0.0	0.0	1.3	6.7	0.07
**Milk and dairy products**	**291.6**	**206.8**	**66.1**	**155.8**	**246.9**	**366.5**	**677.8**	**13.55**
Milk and yogurt	85.0	99.3	0.0	20.8	68.3	114.7	252.1	3.95
Cheese	206.6	171.6	22.9	88.9	167.4	275.6	530.6	9.60
Fresh cheese	58.5	76.0	0.0	12.0	36.0	72.0	188.9	2.72
Aged cheese	148.1	137.4	5.7	55.5	112.1	203.6	390.0	6.88
**Eggs**	**16.7**	**12.6**	**1.6**	**8.1**	**15.0**	**23.5**	**36.9**	**0.78**
**Fish and seafood**	**129.8**	**160.6**	**14.7**	**55.2**	**100.9**	**159.9**	**344.5**	**6.03**
Fish	98.6	149.7	11.6	38.0	70.3	118.7	270.1	4.58
Preserved and tinned fish	72.2	143.3	0.0	21.3	47.2	78.4	215.6	3.36
Non-piscivorous fish	14.6	17.9	0.0	2.1	9.0	20.3	51.1	0.68
Piscivorous fish	11.8	20.5	0.0	0.3	4.1	14.4	46.3	0.55
Crustaceans and mollusks	31.2	43.3	0.0	2.9	15.4	47.1	105.3	1.45
**All vegetables**	**270.7**	**164.7**	**66.9**	**152.7**	**243**	**348.1**	**590.6**	**12.58**
Leafy vegetables	31.7	27.5	4.2	13.1	24.7	41.6	84.0	1.47
Tomatoes	101.9	110.3	2.9	25.4	66.0	143.4	304.6	4.74
Root vegetables	17.8	21.5	0.9	4.2	10.4	23.1	60.0	0.83
Cabbage	2.4	4.1	0.0	0.0	1.0	3.2	10.1	0.11
Other vegetables	116.9	85.6	21.5	54.9	96.4	160.9	275.3	5.43
**Mushrooms**	**0.6**	**0.9**	**0.0**	**0.1**	**0.3**	**0.9**	**1.9**	**0.03**
**Legumes**	**13.6**	**13.5**	**0.4**	**4.6**	**9.9**	**18.4**	**38.9**	**0.63**
**Potatoes**	**37.9**	**37.4**	**2.8**	**16.5**	**27.8**	**49.4**	**108.1**	**1.76**
**Fresh fruit**	**4.2**	**2.5**	**0.7**	**2.5**	**3.9**	**5.4**	**8.8**	**0.20**
Citrus fruit	2.7	1.7	0.5	1.5	2.4	3.5	5.7	0.13
All other fruit	1.6	1.2	0.0	0.7	1.4	2.0	3.8	0.07
**Dry fruit, nuts and seeds**	**1.9**	**3.4**	**0.0**	**0.2**	**0.3**	**1.8**	**9.4**	**0.09**
Dry fruit	0.4	1.3	0.0	0.0	0.1	0.1	2.8	0.02
Nuts and seeds	1.5	2.9	0.0	0.2	0.2	1.5	8.1	0.07
**Sweets products**	**114.7**	**131.5**	**1.1**	**31.1**	**77.4**	**152.8**	**332.7**	**5.33**
Sugar, non-chocolate confectionery	0.4	0.7	0.0	0.0	0.2	0.4	1.5	0.02
Chocolate, candy bars, etc.	2.1	3.4	0.0	0.0	0.7	2.2	8.8	0.10
Ice-cream	4.1	4.6	0.0	0.7	3.1	5.9	12.7	0.19
Cakes, pies and pastries	74.0	114.4	0.0	7.0	30.2	110.3	253.1	3.44
Biscuits, dry cakes	34.1	42.5	0.0	0.0	15.6	59.6	119.6	1.58
**Oils and fats**	**9.7**	**11.5**	**0.1**	**1.4**	**5.9**	**14.5**	**29.5**	**0.45**
Vegetables fats and oils (non-olive)	0.7	1.6	0.0	0.2	0.4	0.8	2.4	0.03
Olive oil	0.0	0.0	0.0	0.0	0.0	0.0	0.1	0.00
Butter and other animal fats	9.0	11.2	0.0	0.6	5.3	13.4	28.0	0.42
**Beverages**	**19.7**	**25.1**	**2.1**	**5.9**	**11.5**	**22.8**	**64.3**	**0.92**
Coffee and tea	3.2	2.8	0.2	1.6	2.6	4.0	7.0	0.15
Wine	1.8	2.4	0.0	0.0	0.8	3.6	7.2	0.08
Red wine	1.1	1.7	0.0	0.0	0.2	1.6	4.8	0.05
White wine	0.8	1.5	0.0	0.0	0.0	0.7	3.8	0.04
Aperitif wines and beers	4.0	11.6	0.0	0.0	0.6	3.4	19.0	0.19
Spirits and liqueurs	0.1	0.5	0.0	0.0	0.0	0.0	0.8	0.00
Fruit juices	4.9	12.3	0.0	0.0	0.7	4.8	22.4	0.23
Soft drinks	5.7	15.2	0.0	0.0	0.0	4.3	25.7	0.26

^1^ Bold characters relate to main food categories.

**Table 4:** Distribution of potassium daily dietary intake in all subjects (in mg/day) and percentage contribution (%) of each food to total intake.

Food ^1^	Mean	SD	P5	P25	P50	P75	P95	%
**Total intake**	**3367.3**	**1127.9**	**1887.4**	**2611.8**	**3238.8**	**3862.6**	**5508.4**	**100**
**Cereals and cereal products**	**390.6**	**203.3**	**108.4**	**252.9**	**362.0**	**505.2**	**751.0**	**11.60**
Pasta and other grains	145.3	103.6	10.4	73.3	127.5	197.7	344.0	4.31
Rice	8.0	11.0	0.0	1.6	5.1	8.8	28.0	0.24
Bread and rolls	148.0	135.9	0..0	37.6	113.3	205.1	388.5	4.39
Pizza, crackers, and other salty snacks	89.3	64.8	9.5	43.0	80.9	117.5	197.4	2.65
**Meat and meat products**	**574.9**	**316.6**	**140.4**	**355.5**	**531.1**	**735.6**	**1219.4**	**17.07**
Red meat	286.7	192.1	36.5	143.8	256.1	389.7	674.0	8.52
White meat	153.9	137.4	2.3	59.2	124.8	211.2	420.2	4.57
Processed meat	127.6	107.8	11.3	53.2	100.0	168.4	337.0	3.79
Offal	6.6	16.6	0.0	0.0	0.0	5.8	27.0	0.20
**Milk and dairy products**	**391.9**	**409.8**	**21.3**	**123.9**	**318.5**	**508.1**	**1046.4**	**11.64**
Milk and yogurt	348.3	402.9	0.0	82.5	281.7	472.8	1031.5	10.34
Cheese	43.6	37.0	4.7	19.1	34.9	56.1	109.0	1.29
Fresh cheese	18.1	23.6	0.0	3.7	11.2	22.4	58.6	0.54
Aged cheese	25.4	23.6	1.0	9.5	19.3	35.0	67.0	0.76
**Eggs**	**18.0**	**13.6**	**1.7**	**8.7**	**16.1**	**25.3**	**39.6**	**0.53**
**Fish and seafood**	**107.3**	**86.2**	**13.7**	**49.7**	**87.5**	**136.4**	**280.0**	**3.19**
Fish	95.7	80.0	9.3	42.1	78.5	122.4	243.4	2.84
Preserved and tinned fish	31.5	37.7	0.0	9.3	23.4	40.0	86.8	0.94
Non-piscivorous fish	37.7	45.0	0.0	5.6	23.8	53.0	143.4	1.12
Piscivorous fish	26.5	37.7	0.0	1.6	13.2	39.9	92.2	0.79
Crustaceans and mollusks	11.6	16.2	0.0	1.1	5.7	17.8	38.2	0.35
**All vegetables**	**506.8**	**291.2**	**143.3**	**308.2**	**453.8**	**639.7**	**1118.6**	**15.05**
Leafy vegetables	102.8	86.0	9.7	40.8	78.1	140.2	284.0	3.05
Tomatoes	234.2	180.4	20.2	105.1	198.4	315.0	572.0	6.95
Root vegetables	87.2	89.4	10.0	30.8	61.2	105.1	254.2	2.59
Cabbage	13.1	22.2	0.0	0.0	5.7	17.3	55.0	0.39
Other vegetables	69.6	53.1	10.1	31.1	56.4	97.0	173.7	2.07
**Mushrooms**	**12.9**	**20.2**	**0.0**	**1.5**	**6.1**	**20.4**	**43.8**	**0.38**
**Legumes**	**188.5**	**187.5**	**5.0**	**63.4**	**136.9**	**254.6**	**539.3**	**5.60**
**Potatoes**	**99.5**	**98.2**	**7.3**	**43.4**	**73.0**	**129.7**	**283.8**	**2.95**
**Fresh fruit**	**529.9**	**314.9**	**91.5**	**315.2**	**494.3**	**696.4**	**1117.6**	**15.74**
Citrus fruit	421.8	264.4	76.6	236.5	383.4	555.7	909.7	12.53
All other fruit	108.1	85.7	1.5	46.4	97.1	139.3	260.0	3.21
**Dry fruit, nuts and seeds**	**12.2**	**21.5**	**0.0**	**1.4**	**2.2**	**11.9**	**60.5**	**0.36**
Dry fruit	3.3	9.2	0.0	0.0	0.8	0.8	20.0	0.10
Nuts and seeds	8.9	17.7	0.0	1.4	1.4	9.0	49.3	0.27
**Sweets products**	**169.4**	**147.5**	**15.0**	**71.9**	**133.5**	**221.9**	**429.5**	**5.03**
Sugar, non-chocolate confectionery	27.6	33.4	0.0	6.2	18.6	41.4	80.0	0.82
Chocolate, candy bars, etc.	39.6	65.8	0.0	0.0	12.8	42.9	169.4	1.18
Ice-cream	20.8	23.3	0.0	3.7	15.5	29.5	64.0	0.62
Cakes, pies and pastries	53.2	82.2	0.0	5.0	21.7	79.3	181.9	1.58
Biscuits, dry cakes	28.2	35.2	0.0	0.0	12.9	49.3	99.0	0.84
**Oils and fats**	**1.1**	**1.1**	**0.1**	**0.4**	**0.8**	**1.6**	**3.1**	**0.03**
Vegetables fats and oils (non-olive)	0.1	0.1	0.0	0.0	0.0	0.1	0.2	0.002
Olive oil	0.1	0.1	0.0	0.1	0.1	0.2	0.3	0.004
Butter and other animal fats	0.9	1.1	0.0	0.2	0.6	1.4	2.8	0.03
**Beverages**	**364.4**	**274.9**	**67.8**	**193.8**	**310.8**	**473.0**	**799.5**	**10.82**
Coffee and tea	180.8	162.2	4.0	83.4	162.5	231.6	394.3	5.37
Wine	73.3	97.1	0.0	0.8	29.7	108.4	265.6	2.18
Red wine	52.8	84.3	0.0	0.0	11.3	80.4	236.0	1.57
White wine	20.6	40.7	0.0	0.0	0.8	19.2	99.8	0.61
Aperitif wines and beers	27.2	78.7	0.0	0.0	3.7	26.7	127.8	0.81
Spirits and liqueurs	0.2	0.7	0.0	0.0	0.0	0.0	1.0	0.01
Fruit juices	75.1	153.6	0.0	0.0	17.5	81.1	351.0	2.23
Soft drinks	7.8	20.8	0.0	0.0	0.0	5.8	35.1	0.23

^1^ Bold characters relate to main food categories.

**Table 5:** Distribution of sodium and potassium daily dietary intake below (<4) and above (≥4) the median adherence to the Italian Mediterranean Index (IMI) (in mg/day). Mean, standard deviation and *p* values form the *t*-test of difference between means are reported.

	Sodium			Potassium		
Food ^1^	IMI < 4 N = 275	IMI ≥ 4 N = 444	*p* Value	IMI < 4 N = 275	IMI ≥ 4 N = 444	*p* Value
**Total intake**	**2164.7 (849.5)**	**2143.8 (880.0)**	**0.755**	**3049.6 (901.6)**	**3564.1 (1207.3)**	**<0.001**
**Cereals and cereal products**	**731.5 (465.3)**	**703.7 (446.2)**	**0.424**	**384.7 (202.9)**	**394.2 (203.6)**	**0.542**
Pasta and other grains	28.6 (21.0)	33.3 (23.1)	0.006	131.8 (96.8)	153.6 (106.8)	0.006
Rice	0.04 (0.05)	0.05 (0.07)	0.046	7.0 (9.3)	8.7 (12.0)	0.046
Bread and rolls	444.4 (415.6)	411.1 (372.1)	0.265	155.1 (145.1)	143.5 (129.9)	0.265
Pizza, crackers, and other salty snacks	258.5 (173.7)	259.3 (201.7)	0.959	90.7 (60.1)	88.4 (67.6)	0.645
**Meat and meat products**	**601.8 (361.9)**	**479.8 (334.1)**	**<0.001**	**624.3 (294.0)**	**544.4 (326.5)**	**0.001**
Red meat	130.4 (78.8)	103.7 (77.4)	<0.001	327.9 (191.3)	261.2 (188.4)	<0.001
White meat	44.1 (38.6)	50.5 (44.9)	0.049	141.0 (123.2)	162.0 (145.0)	0.046
Processed meat	426.0 (335.2)	323.9 (280.7)	<0.001	149.8 (117.9)	113.9 (98.7)	<0.001
Offal	1.3 (3.4)	1.7 (4.1)	0.180	5.6 (14.7)	7.3 (17.7)	0.180
**Milk and dairy products**	**281.2 (195.8)**	**298.1 (213.4)**	**0.289**	**345.2 (397.5)**	**420.8 (415.1)**	**0.016**
Milk and yogurt	73.5 (96.7)	92.1 (100.3)	0.015	301.3 (392.1)	377.4 (407.1)	0.014
Cheese	207.7 (163.8)	205.9 (176.4)	0.896	43.9 (35.0)	43.4 (38.2)	0.856
Fresh cheese	59.5 (67.3)	57.9 (80.9)	0.787	18.4 (20.9)	18.0 (25.1)	0.787
Aged cheese	148.2 (129.6)	148.1 (142.2)	0.989	25.5 (22.3)	25.4 (24.4)	0.989
**Eggs**	**16.2 (11.3)**	**17.0 (13.4)**	**0.393**	**17.4 (12.1)**	**18.3 (14.4)**	**0.393**
**Fish and seafood**	**117.6 (211.0)**	**137.3 (119.0)**	**0.110**	**87.8 (72.2)**	**119.3 (91.8)**	**<0.001**
Fish	89.5 (204.4)	104.2 (102.1)	0.202	77.3 (66.9)	107.0 (85.2)	<0.001
Preserved and tinned fish	69.7 (201.6)	73.8 (90.3)	0.713	28.9 (43.1)	33.1 (33.8)	0.141
Non-piscivorous fish	11.3 (13.3)	16.6 (20.0)	<0.001	28.2 (30.8)	43.5 (51.1)	<0.001
Piscivorous fish	8.4 (13.9)	13.8 (23.5)	<0.001	20.2 (29.7)	30.4 (41.5)	<0.001
Crustaceans and mollusks	28.1 (35.5)	33.1 (47.3)	0.127	10.5 (13.5)	12.3 (17.6)	<0.001
**All vegetables**	**204.7 (104.5)**	**311.6 (181.3)**	**<0.001**	**358.6 (168.8)**	**598.5 (312.6)**	**<0.001**
Leafy vegetables	23.0 (17.7)	37.1 (30.9)	<0.001	68.8 (56.0)	123.8 (94.2)	<0.001
Tomatoes	80.2 (76.4)	115.4 (125.0)	<0.001	174.4 (119.3)	271.2 (200.8)	<0.001
Root vegetables	10.7 (11.3)	22.2 (24.9)	<0.001	56.9 (49.8)	105.9 (102.4)	<0.001
Cabbage	1.5 (2.5)	3.0 (4.7)	<0.001	8.3 (13.7)	16.0 (25.7)	<0.001
Other vegetables	89.3 (59.4)	133.9 (94.5)	<0.001	50.3 (35.9)	81.5 (58.3)	<0.001
**Mushrooms**	**0.5 (0.6)**	**0.6 (1.0)**	**0.073**	**11.2 (14.2)**	**13.9 (23.1)**	**0.073**
**Legumes**	**9.9 (9.4)**	**15.9 (15.1)**	**<0.001**	**136.5 (130.8)**	**220.7 (209.0)**	**<0.001**
**Potatoes**	**38.4 (26.3)**	**37.5 (42.9)**	**0.756**	**100.9 (69.1)**	**98.6 (112.6)**	**0.756**
**Fresh fruit**	**3.3 (1.9)**	**4.8 (2.6)**	**<0.001**	**407.7 (234.6)**	**605.6 (334.2)**	**<0.001**
Citrus fruit	2.0 (1.2)	3.0 (1.8)	<0.001	320.7 (192.8)	484.5 (283.0)	<0.001
All other fruit	1.3 (1.1)	1.7 (1.3)	<0.001	87.0 (73.6)	121.1 (90.0)	<0.001
**Dry fruit, nuts and seeds**	**1.9 (3.7)**	**1.9 (3.2)**	**0.861**	**12.0 (23.2)**	**12.3 (20.5)**	**0.844**
Dry fruit	0.4 (1.2)	0.5 (1.3)	0.674	3.1 (8.4)	3.4 (9.7)	0.674
Nuts and seeds	1.5 (3.2)	1.5 (2.7)	0.983	8.9 (19.7)	8.9 (16.4)	0.983
**Sweets products**	**123.7 (142.2)**	**109.1 (124.2)**	**0.149**	**181.1 (151.4)**	**162.2 (144.8)**	**0.095**
Sugar, non-chocolate confectionery	0.4 (0.7)	0.3 (0.8)	0.338	29.2 (33.2)	26.6 (33.6)	0.320
Chocolate, candy bars, etc.	2.2 (3.6)	2.0 (3.3)	0.322	42.7 (69.5)	37.7 (63.5)	0.322
Ice-cream	4.3 (4.4)	4.0 (4.8)	0.380	21.7 (21.9)	20.1 (24.1)	0.380
Cakes, pies and pastries	84.2 (125.8)	67.7 (106.4)	0.060	60.5 (90.5)	48.7 (76.5)	0.060
Biscuits, dry cakes	32.5 (42.1)	35.1 (42.8)	0.436	26.9 (34.8)	29.0 (35.4)	0.436
**Oils and fats**	**11.6 (13.0)**	**8.6 (10.2)**	**<0.001**	**1.3 (1.3)**	**1.0 (1.0)**	**<0.001**
Vegetables fats and oils (non-olive)	0.8 (1.6)	0.7 (1.6)	0.658	0.1 (0.1)	0.1 (0.1)	0.709
Olive oil	0.02 (0.01)	0.02 (0.02)	<0.001	0.1 (0.0)	0.2 (0.1)	<0.001
Butter and other animal fats	10.8 (12.9)	7.9 (9.8)	<0.001	1.2 (1.3)	0.8 (0.9)	<0.001
**Beverages**	**22.6 (24.0)**	**17.9 (25.7)**	**0.016**	**380.8 (269.1)**	**354.2 (278.3)**	**0.208**
Coffee and tea	3.2 (2.7)	3.2 (2.8)	0.993	181.5 (183.0)	180.3 (148.0)	0.924
Wine	2.3 (2.5)	1.6 (2.3)	<0.001	87.2 (99.7)	64.7 (94.5)	0.003
Red wine	1.2 (1.7)	1.0 (1.7)	0.087	59.6 (86.5)	48.5 (82.8)	0.087
White wine	1.0 (1.8)	0.6 (1.3)	<0.001	27.6 (48.1)	16.2 (34.8)	<0.001
Aperitif wines and beers	4.4 (9.8)	3.7 (12.6)	0.465	30.1 (66.4)	25.5 (85.4)	0.449
Spirits and liqueurs	0.2 (0.7)	0.1 (0.4)	0.001	0.3 (0.9)	0.1 (0.5)	0.001
Fruit juices	4.3 (8.1)	5.3 (14.3)	0.297	70.4 (129.6)	78.0 (166.9)	0.521
Soft drinks	8.3 (16.2)	4.1 (14.3)	<0.001	11.3 (22.1)	5.6 (19.6)	<0.001

^1^ Bold characters relate to main food categories.

**Table 6:** Distribution of sodium and potassium daily dietary intake below (<7.5) and above (≥7.5) the median adherence to the Mediterranean–DASH Diet Intervention for Neurodegenerative Delay (MIND) (in mg/day). Mean, standard deviation, and *p* values from the *t*-test of differences between means are reported.

	Sodium			Potassium		
Food ^1^	MIND < 7.5 N = 288	MIND ≥ 7.5 N = 431	*p* Value	MIND < 7.5 N = 288	MIND ≥ 7.5 N = 431	*p* Value
**Total intake**	**2121.5 (841.8)**	**2172.1 (885.4)**	**0.444**	**3058.1 (993.1)**	**3573.9 (1165.8)**	**<0.001**
**Cereals and cereal products**	**687.3 (440.1)**	**732.3 (461.9)**	**0.193**	**390.3 (194.9)**	**390.7 (208.9)**	**0.981**
Pasta and other grains	33.4 (22.9)	30.2 (22.1)	0.057	154.3 (105.6)	139.3 (101.8)	0.057
Rice	0.04 (0.06)	0.05 (0.06)	0.160	7.3 (10.1)	8.5 (11.6)	0.160
Bread and rolls	406.9 (375.3)	435.1 (398.5)	0.341	142.0 (131.0)	151.9 (139.1)	0.341
Pizza, crackers, and other salty snacks	247.0 (190.8)	267.0 (191.5)	0.170	86.7 (67.6)	91.1 (62.9)	0.380
**Meat and meat products**	**595.0 (354.1)**	**480.7 (339.6)**	**<0.001**	**607.0 (298.2)**	**553.5 (327.0)**	**0.026**
Red meat	123.6 (77.8)	107.4 (79.2)	0.007	321.2 (193.7)	263.7 (187.8)	<0.001
White meat	39.5 (38.1)	53.8 (44.7)	<0.001	127.0 (123.5)	171.9 (143.2)	<0.001
Processed meat	430.1 (326.9)	318.1 (283.7)	<0.001	151.3 (115.0)	111.9 (99.8)	<0.001
Offal	1.8 (4.6)	1.4 (3.3)	0.202	7.6 (19.7)	6.0 (14.2)	0.202
**Milk and dairy products**	**281.8 (189.9)**	**298.2 (217.4)**	**0.299**	**342.1 (376.8)**	**425.2 (427.7)**	**0.008**
Milk and yogurt	72.9 (92.1)	93.1 (103.1)	0.007	298.3 (372.1)	381.7 (419.3)	0.007
Cheese	208.9 (162.7)	205.1 (177.4)	0.769	43.7 (34.2)	43.5 (38.8)	0.927
Fresh cheese	56.7 (66.3)	59.6 (81.8)	0.616	17.6 (20.6)	18.5 (25.4)	0.616
Aged cheese	152.2 (134.7)	145.4 (139.3)	0.520	26.1 (23.1)	25.0 (23.9)	0.520
**Eggs**	**16.0 (12.4)**	**17.2 (12.8)**	**0.210**	**17.2 (13.3)**	**18.5 (13.7)**	**0.210**
**Fish and seafood**	**100.8 (96.8)**	**149.1 (189.5)**	**<0.001**	**81.4 (66.9)**	**124.6 (93.1)**	**<0.001**
Fish	75.3 (84.5)	114.1 (179.1)	0.001	71.8 (62.3)	111.6 (86.4)	<0.001
Preserved and tinned fish	55.2 (75.9)	83.6 (173.6)	0.009	24.4 (24.1)	36.3 (43.8)	<0.001
Non-piscivorous fish	11.1 (14.1)	16.9 (19.8)	<0.001	28.1 (35.8)	44.0 (49.3)	<0.001
Piscivorous fish	9.0 (16.3)	13.6 (22.8)	0.003	19.3 (28.2)	31.3 (42.3)	<0.001
Crustaceans and mollusks	25.6 (32.9)	35.0 (48.6)	0.004	9.6 (12.5)	13.0 (18.1)	0.006
**All vegetables**	**222.0 (146.2)**	**303.2 (168.5)**	**<0.001**	**405.1 (262.6)**	**574.7 (289.8)**	**<0.001**
Leafy vegetables	22.4 (21.1)	37.9 (29.5)	<0.001	72.1 (73.7)	123.2 (87.6)	<0.001
Tomatoes	95.0 (99.9)	106.6 (116.6)	0.169	208.0 (168.8)	251.7 (185.9)	0.001
Root vegetables	12.4 (16.6)	21.4 (23.6)	<0.001	66.5 (72.3)	101.0 (96.8)	<0.001
Cabbage	1.4 (2.3)	3.1 (4.8)	<0.001	7.6 (12.3)	16.7 (26.3)	<0.001
Other vegetables	90.8 (74.2)	134.3 (88.3)	<0.001	50.9 (45.1)	82.1 (54.4)	<0.001
**Mushrooms**	**0.4 (0.7)**	**0.6 (1.0)**	**0.002**	**10.0 (15.7)**	**14.8 (22.5)**	**0.002**
**Legumes**	**9.4 (10.6)**	**16.4 (14.5)**	**<0.001**	**130.9 (147.4)**	**227.0 (201.2)**	**<0.001**
**Potatoes**	**35.0 (34.5)**	**39.8 (39.1)**	**0.093**	**91.9 (90.5)**	**104.5 (102.8)**	**0.093**
**Fresh fruit**	**3.4 (2.1)**	**4.8 (2.6)**	**<0.001**	**423.7 (260.7)**	**600.9 (328.1)**	**<0.001**
Citrus fruit	2.1 (1.3)	3.0 (1.8)	<0.001	332.8 (211.2)	481.3 (279.5)	<0.001
All other fruit	1.3 (1.1)	1.7 (1.3)	<0.001	90.9 (77.7)	119.6 (88.9)	<0.001
**Dry fruit, nuts and seeds**	**1.1 (2.2)**	**2.4 (3.9)**	**<0.001**	**7.3 (13.9)**	**15.5 (24.9)**	**<0.001**
Dry fruit	0.2 (0.8)	0.6 (1.5)	<0.001	1.7 (5.9)	4.3 (10.7)	<0.001
Nuts and seeds	0.9 (1.9)	1.8 (3.3)	<0.001	5.6 (11.9)	11.2 (20.4)	<0.001
**Sweets products**	**137.3 (149.3)**	**99.6 (115.8)**	**<0.001**	**186.6 (152)**	**157.9 (143.5)**	**0.011**
Sugar, non-chocolate confectionery	0.4 (1.0)	0.3 (0.5)	<0.010	26.9 (25.6)	28.1 (37.8)	0.636
Chocolate, candy bars, etc.	2.1 (3.5)	2.1 (3.4)	0.996	39.6 (67.4)	39.6 (64.9)	0.996
Ice-cream	4.4 (5.1)	3.9 (4.3)	0.180	22.2 (25.5)	19.8 (21.6)	0.180
Cakes, pies and pastries	91.5 (137.2)	62.4 (94.6)	<0.001	65.8 (98.6)	44.8 (68.0)	0.001
Biscuits, dry cakes	38.9 (41.9)	30.9 (42.7)	0.014	32.2 (34.6)	25.6 (35.3)	0.014
**Oils and fats**	**11.2 (11)**	**8.8 (11.7)**	**0.006**	**1.3 (1.1)**	**1.0 (1.1)**	**0.004**
Vegetables fats and oils (non-olive)	0.9 (2.1)	0.6 (1.1)	0.029	0.06 (0.14)	0.05 (0.08)	0.035
Olive oil	0.02 (0.01)	0.03 (0.02)	<0.001	0.11 (0.07)	0.14 (0.08)	<0.001
Butter and other animal fats	10.2 (10.7)	8.1 (11.5)	0.012	1.1 (1.1)	0.8 (1.1)	0.001
**Beverages**	**20.6 (23.0)**	**19.1 (26.4)**	**0.410**	**363.3 (252.6)**	**365.2 (289.2)**	**0.930**
Coffee and tea	3.0 (2.2)	3.3 (3.1)	0.108	160.4 (124.9)	194.4 (181.8)	0.006
Wine	2.4 (2.8)	1.5 (2.1)	<0.001	92.9 (112.9)	60.3 (82.5)	<0.001
Red wine	1.3 (2.0)	0.9 (1.4)	0.001	65.9 (100.9)	44.0 (69.9)	0.001
White wine	1.0 (1.8)	0.6 (1.3)	0.001	27.0 (47.9)	16.3 (34.6)	0.001
Aperitif wines and beers	4.4 (10.6)	3.7 (12.3)	0.423	30.3 (72.3)	25.2 (82.7)	0.400
Spirits and liqueurs	0.2 (0.6)	0.1 (0.4)	0.041	0.2 (0.8)	0.1 (0.6)	0.041
Fruit juices	4.2 (7.9)	5.3 (14.6)	0.212	70.6 (131.9)	78.2 (166.7)	0.516
Soft drinks	6.6 (14.1)	5.1 (15.9)	0.210	8.9 (19.3)	7.0 (21.6)	0.210

^1^ Bold characters relate to main food categories.

## Data Availability

The data presented in this study are available on reasonable request from the corresponding author. The data are not publicly available due to privacy reasons.

## Supplementary Materials

The following are available online at https://www.mdpi.com/article/10.3390/nu13082681/s1, Table S1: Food intake (g/day) according to different categories for the whole study population and by sex. Mean and standard deviation (SD) along with *p* values from *t*-test for independent samples for the difference between men and women are reported. Table S2: Distribution of sodium daily dietary intake in men (in mg/day) and percentage contribution (%) of each food to total intake. Table S3: Distribution of sodium daily dietary intake in women (in mg/day) and percentage contribution (%) of each food to total intake. Table S4: Distribution of potassium daily dietary intake in men (in mg/day) and percentage contribution (%) of each food to total intake. Supplemental Table S5: Distribution of potassium daily dietary intake in women (in mg/day) and percentage contribution (%) of each food to total intake.
