# Supplementary material for: Sodium and Potassium Content of Foods Consumed in an Italian Population and the Impact of Adherence to a Mediterranean Diet on Their Intake
Source: Nutrients. 2021 Aug 1;13(8):2681. doi: 10.3390/nu13082681 (PMC8401684; doi:10.3390/nu13082681)
Supplement: Supplementary file 1 [file nutrients-13-02681-s001.zip › nutrients-1277889-supplementary.pdf]

nutrients

SUPPLEMENTAL MATERIAL

MDPI

Supplemental Table S1. Food intake (g/day) according to different categories for the whole study population and by sex. Mean and standard deviation (SD) along with P values from t-test for independent samples for the difference between men and women are reported.

Foods[1]	All (N=719)	Men (N=319)	Women (N=400)	*P value*
	**Mean (SD)**	**Mean (SD)**	**Mean (SD)**	
**Cereals and cereal products**	**188.5 (99.3)**	**206.4 (104.6)**	**174.2 (92.5)**	**<0.001**
Pasta, other grain	57.1 (40.7)	70.1 (45.3)	46.8 (33.2)	<0.001
Rice	5.6 (7.6)	6.2 (8.7)	5.0 (6.7)	0.031
Bread	78.3 (72.0)	83.9 (73.4)	73.9 (70.6)	0.063
Crackers, crispbread, salty snacks	47.5 (34.2)	46.2 (35.7)	48.5 (33.0)	0.371
**Meat and meat products**	**128.4 (70.9)**	**142.4 (73.5)**	**117.2 (66.8)**	**<0.001**
Red meat	67.4 (45.1)	76.8 (49.6)	59.9 (39.7)	0.001
White meat	29.7 (26.2)	31.2 (25.6)	28.4 (26.6)	0.154
Processed meat	29.5 (24.9)	32.3 (27.2)	27.3 (22.7)	0.007
Offal	1.8 (4.6)	2.1 (5.2)	1.6 (4.0)	0.146
**Milk and dairy products**	**229.4 (216.1)**	**201.8 (191.7)**	**251.5 (231.6)**	**0.002**
Milk and yogurt	188.9 (209.3)	159.4 (188.3)	212.5 (222.0)	0.001
Cheese	40.5 (33.9)	42.4 (35.5)	39.0 (32.5)	0.184
Fresh cheese	14.6 (19.0)	11.9 (15.5)	16.8 (21.1)	<0.001
Aged cheese	25.9 (24.0)	30.5 (27.6)	22.2 (20.1)	<0.001
**Eggs**	**15.1 (11.4)**	**14.6 (11.2)**	**15.4 (11.5)**	**0.316**
**Fish and seafood**	**35.1 (28.1)**	**35.5 (26.8)**	**34.9 (29.0)**	**0.780**
Fish	27.9 (23.1)	28.5 (22.6)	27.5 (23.6)	0.502
Preserved and tinned fish	9.3 (10.7)	10.2 (9.7)	8.5 (11.4)	0.034
Non-piscivorous fish	10.4 (12.3)	10.8 (13.1)	10.2 (11.6)	0.507
Piscivorous fish	8.2 (11.7)	7.5 (10.7)	8.7 (12.4)	0.189
Crustaceans and molluscs	7.2 (10.1)	7.0 (9.4)	7.4 (10.6)	0.566
**All vegetables**	**158.2 (93.4)**	**154.1 (87.2)**	**161.4 (98.1)**	**0.296**
Leafy vegetables	31.6 (25.6)	29.4 (22.8)	33.3 (27.5)	0.038
Other vegetables	27.3 (20.0)	24.0 (17.0)	29.9 (21.8)	<0.001
Tomatoes	63.4 (50.8)	67.0 (52.0)	60.5 (49.7)	0.090
Root vegetables	31.8 (32.4)	29.9 (30.3)	33.3 (33.9)	0.163
Cabbage	4.2 (7.1)	3.8 (8.0)	4.4 (6.2)	0.269
**Mushrooms**	**2.5 (4.0)**	**2.5 (4.2)**	**2.5 (3.8)**	**0.911**
**Legumes**	**18.7 (18.6)**	**19.5 (19.3)**	**18.1 (18.1)**	**0.334**
**Potatoes**	**24.5 (24.2)**	**25.5 (26.4)**	**23.7 (22.3)**	**0.322**
**Fresh fruits**	**279.4 (165.7)**	**270.3 (162.8)**	**286.7 (167.8)**	**0.187**
Citrus fruits	216.3 (135.6)	207.0 (132.9)	223.8 (137.5)	0.100
All other fruits	63.1 (50.0)	63.3 (49.5)	62.9 (50.5)	0.927
**Dry fruits, nuts and seeds**	**1.7 (3.0)**	**1.8 (2.9)**	**1.6 (3.0)**	**0.295**
Dry fruits	0.4 (1.1)	0.5 (1.2)	0.3 (1.0)	0.155
Nuts and seeds	1.3 (2.5)	1.3 (2.3)	1.2 (2.8)	0.543
**Sweets, chocolate, cakes, etc.**	**86.5 (73.8)**	**82.9 (77.3)**	**89.4 (70.8)**	**0.243**
Sugar, confectionery not chocolate	18.5 (22.5)	17.3 (18.2)	19.4 (25.4)	0.220
Chocolate, candy bars, etc.	5.3 (8.7)	4.9 (8.5)	5.6 (8.9)	0.287
Ice-cream	13.9 (15.6)	13.2 (16.9)	14.2 (14.5)	0.413
Cakes, pies and pastries	35.1 (54.3)	34.0 (61.6)	36.0 (47.7)	0.620
Biscuits, dry cakes	13.7 (17.1)	13.1 (17.2)	14.2 (17.1)	0.598
**Oils and fats**	**27.2 (13.5)**	**27.9 (12.7)**	**26.7 (14.1)**	**0.251**
Vegetable fats and oils (not olive)	2.4 (5.7)	2.6 (6.1)	2.2 (5.4)	0.321
Olive oil	22.0 (12.6)	22.0 (12.1)	22.1 (12.9)	0.893
Butter and other animal fats	2.8 (3.5)	3.3 (4.1)	2.4 (2.9)	0.001
**Beverages**	**429.5 (341.3)**	**479.5 (322.5)**	**389.7 (350.8)**	**<0.001**
Coffee and tea	148.4 (160.3)	120.6 (109.3)	170.6 (188.7)	<0.001
Wines	126.0 (164.1)	191.4 (190.0)	73.9 (116.3)	<0.001
Red wine	74.5 (119.0)	116.6 (144.1)	41.0 (80.0)	<0.001
White wine	51.5 (102.0)	74.8 (123.8)	32.9 (75.8)	<0.001
Aperitif wines and beers	41.4 (117.5)	49.9 (123.8)	34.6 (126.0)	0.082
Spirits and liqueurs	3.1 (11.0)	5.3 (14.9)	1.3 (5.7)	<0.001
Fruit juices	66.4 (135.0)	67.6 (126.7)	65.5 (141.4)	0.834
Soft drinks	44.2 (118.2)	44.7 (100.7)	43.8 (130.6)	0.916

[1]Bold characters relate to main food categories

Supplemental Table S2. Distribution of sodium daily dietary intake in men (in mg/day) and percentage contribution (%) of each food to total intake.

**Food**[1]	**Mean**	**SD**	**P5**	**P25**	**P50**	**P75**	**P95**	**%**
Total intake	***2246.6***	***885.6***	***1038.8***	***1662.1***	***2075.8***	***2777.8***	***3833.3***	***100***
**Cereals and cereal products**	**744.7**	**477.2**	**108.3**	**385.8**	**684.8**	**1018.2**	**1637.4**	**33.15**
Pasta and other grains	38.6	25.0	2.8	22.2	35.2	50.0	81.9	1.72
Rice	0.0	0.1	0.0	0.0	0.0	0.1	0.2	0.00
Bread and rolls	454.0	397.1	0.0	96.8	385.2	657.8	1246.4	20.21
Pizza, crackers, and other salty snacks	252.0	204.4	24.0	113.4	210.9	339.6	583.7	11.22
**Meat and meat products**	**575.7**	**375.5**	**130.1**	**326.5**	**496.5**	**718.3**	**1331.6**	**25.63**
Red meat	125.7	83.2	17.1	65.5	112.2	169.4	287.1	5.60
White meat	51.0	42.1	1.7	21.7	41.9	71.5	133.1	2.27
Processed meat	397.2	334.4	40.6	166.2	304.0	533.0	1064.8	17.68
Offal	1.8	4.4	0.0	0.0	0.0	1.7	6.7	0.08
**Milk and dairy products**	**292.7**	**205.7**	**65.8**	**151.6**	**249.8**	**385.6**	**675.8**	**13.03**
Milk and yogurt	70.7	86.0	0.0	4.8	51.2	100.0	205.8	3.15
Cheese	222.0	186.2	29.2	92.3	182.4	293.6	558.0	9.88
Fresh cheese	47.6	62.0	0.0	4.8	30.4	64.4	162.1	2.12
Aged cheese	174.4	157.6	18.3	66.3	132.7	240.8	455.2	7.76
**Eggs**	**16.2**	**12.5**	**1.4**	**7.8**	**13.8**	**22.2**	**39.5**	**0.72**
**Fish and seafood**	**134.5**	**109.4**	**14.1**	**60.7**	**111.1**	**169.6**	**337.5**	**5.99**
Fish	104.3	95.5	13.7	42.9	81.3	135.4	273.8	4.64
Preserved and tinned fish	78.9	84.8	0.0	28.7	60.3	99.3	240.0	3.51
Non-piscivorous fish	14.6	18.3	0.0	1.9	8.1	20.1	55.5	0.65
Piscivorous fish	10.9	18.4	0.0	0.3	3.5	12.3	46.3	0.49
Crustaceans and mollusks	30.1	40.6	0.0	2.9	14.7	49.2	92.5	1.34
**All vegetables**	**280.4**	**174.3**	**74.6**	**154.6**	**247.6**	**352.6**	**642.8**	**12.48**
Leafy vegetables	29.0	22.7	3.2	12.4	23.1	39.0	73.3	1.29
Tomatoes	129.1	130.4	3.6	40.8	93.6	180.4	369.6	5.75
Root vegetables	14.6	18.5	0.8	3.4	8.2	19.0	48.7	0.65
Cabbage	2.2	4.7	0.0	0.0	0.8	2.4	10.1	0.10
Other vegetables	105.4	73.9	18.8	49.2	86.8	146.0	253.6	4.69
**Mushrooms**	**0.5**	**0.9**	**0.0**	**0.0**	**0.3**	**0.9**	**1.9**	**0.02**
**Pulses**	**14.1**	**14.0**	**0.5**	**4.5**	**10.3**	**18.7**	**41.9**	**0.63**
**Potatoes**	**39.4**	**40.8**	**2.6**	**16.5**	**27.9**	**47.5**	**118.4**	**1.75**
**Fresh fruit**	**4.1**	**2.5**	**0.6**	**2.5**	**3.8**	**5.3**	**9.3**	**0.18**
Citrus fruit	2.5	1.6	0.4	1.4	2.3	3.3	5.9	0.11
All other fruit	1.6	1.2	0.0	0.7	1.4	2.1	3.8	0.07
**Dry fruit, nuts and seeds**	**2.1**	**3.3**	**0.0**	**0.2**	**0.3**	**2.0**	**9.5**	**0.09**
Dry fruit	0.5	1.4	0.0	0.0	0.1	0.1	3.2	0.02
Nuts and seeds	1.5	2.6	0.0	0.2	0.2	1.6	8.1	0.07
**Sweets products**	**110.6**	**141.1**	**0.1**	**24.2**	**71.1**	**152.6**	**331.1**	**4.92**
Sugar, non-chocolate confectionery	0.4	0.6	0.0	0.0	0.2	0.4	1.4	0.02
Chocolate, candy bars, etc.	1.9	3.3	0.0	0.0	0.5	2.2	8.8	0.08
Ice-cream	4.0	5.0	0.0	0.7	2.3	5.9	14.3	0.18
Cakes, pies and pastries	71.7	129.8	0.0	0.0	26.6	99.3	246.1	3.19
Biscuits, dry cakes	32.7	42.7	0.0	0.0	12.7	59.6	119.1	1.46
**Oils and fats**	**11.1**	**11.8**	**0.2**	**1.6**	**7.6**	**16.9**	**33.0**	**0.49**
Vegetables fats and oils (non-olive)	0.8	1.9	0.0	0.1	0.4	0.8	4.2	0.04
Olive oil	0.0	0.0	0.0	0.0	0.0	0.0	0.1	0.00
Butter and other animal fats	10.2	11.5	0.0	1.2	6.5	15.8	31.0	0.45
**Beverages**	**20.6**	**22.6**	**2.4**	**7.1**	**12.4**	**23.4**	**67.2**	**0.92**
Coffee and tea	2.7	2.1	0.0	1.6	2.4	3.5	5.7	0.12
Wine	2.8	2.8	0.0	0.3	1.8	3.8	7.4	0.12
Red wine	1.7	2.1	0.0	0.0	0.8	3.2	5.4	0.08
White wine	1.1	1.9	0.0	0.0	0.3	1.7	4.7	0.05
Aperitif wines and beers	4.8	10.4	0.0	0.0	0.6	4.7	23.6	0.21
Spirits and liqueurs	0.2	0.7	0.0	0.0	0.0	0.1	1.8	0.01
Fruit juices	4.3	10.0	0.0	0.0	0.5	4.0	22.4	0.19
Soft drinks	5.8	13.0	0.0	0.0	0.0	7.3	25.7	0.26

[1]Bold characters relate to main food categories

Supplemental Table S3. Distribution of sodium daily dietary intake in women (in mg/day) and percentage contribution (%) of each food to total intake.

Food[1]	Mean	SD	P5	P25	P50	P75	P95	%
Total intake	*2076.2*	*847.1*	*991.2*	*1527.3*	*1981.7*	*2405.9*	*3734.9*	*100*
**Cereals and cereal products**	**690.1**	**432.8**	**139.7**	**405.8**	**648.9**	**900.4**	**1413.7**	**33.24**
Pasta and other grains	25.8	18.3	1.8	13.3	22.6	35.5	60.2	1.24
Rice	0.0	0.1	0.0	0.0	0.0	0.0	0.1	0.00
Bread and rolls	399.7	381.8	0.0	116.6	309.4	558.6	1079.8	19.25
Pizza, crackers, and other salty snacks	264.6	180.3	34.9	138.6	228.6	360.9	578.5	12.74
**Meat and meat products**	**487.2**	**323.0**	**108.8**	**275.2**	**414.0**	**632.6**	**1091.8**	**23.47**
Red meat	104.5	74.2	7.3	52.3	89.4	142.8	249.6	5.03
White meat	45.7	43.0	0.0	17.4	32.9	63.1	128.8	2.20
Processed meat	335.7	279.7	27.7	145.9	268.3	441.9	842.0	16.17
Offal	1.3	3.4	0.0	0.0	0.0	0.8	6.7	0.06
**Milk and dairy products**	**290.7**	**208.0**	**68.0**	**160.1**	**241.2**	**356.6**	**680.2**	**14.00**
Milk and yogurt	96.4	107.5	0.0	41.7	72.9	119.3	253.1	4.64
Cheese	194.3	158.1	14.9	88.0	159.8	254.5	481.5	9.36
Fresh cheese	67.2	84.6	0.0	17.6	43.2	85.3	193.7	3.24
Aged cheese	127.1	114.7	2.0	51.5	99.8	170.4	318.8	6.12
**Eggs**	**17.1**	**12.8**	**1.8**	**8.5**	**15.6**	**23.8**	**34.8**	**0.82**
**Fish and seafood**	**126.0**	**192.0**	**16.7**	**52.3**	**91.5**	**145.8**	**362.4**	**6.07**
Fish	94.0	181.8	8.7	36.2	62.5	104.9	265.4	4.53
Preserved and tinned fish	66.9	176.6	0.0	14.1	41.6	65.7	185.1	3.22
Non-piscivorous fish	14.6	17.7	0.0	2.4	9.8	21.0	47.5	0.70
Piscivorous fish	12.5	22.1	0.0	0.4	4.4	15.3	46.3	0.60
Crustaceans and mollusks	32.1	45.3	0.0	3.0	15.7	43.3	141.3	1.55
**All vegetables**	**263.0**	**156.5**	**61.5**	**149.3**	**235.6**	**344.0**	**557.8**	**12.67**
Leafy vegetables	33.8	30.7	4.8	13.9	25.2	43.3	97.9	1.63
Tomatoes	80.3	85.3	1.8	17.9	50.2	116.4	247.5	3.87
Root vegetables	20.3	23.3	1.0	5.0	12.5	26.5	71.8	0.98
Cabbage	2.6	3.6	0.0	0.2	1.2	3.8	10.4	0.13
Other vegetables	126.0	92.9	25.1	60.3	102.6	169.8	300.1	6.07
**Mushrooms**	**0.6**	**0.8**	**0.0**	**0.1**	**0.2**	**0.9**	**1.9**	**0.03**
**Pulses**	**13.2**	**13.1**	**0.4**	**4.6**	**9.8**	**17.4**	**37.1**	**0.64**
**Potatoes**	**36.6**	**34.4**	**3.8**	**15.6**	**27.6**	**49.9**	**97.7**	**1.76**
**Fresh fruit**	**4.3**	**2.5**	**0.8**	**2.5**	**4.2**	**5.6**	**8.7**	**0.21**
Citrus fruit	2.7	1.7	0.5	1.6	2.5	3.6	5.7	0.13
All other fruit	1.6	1.2	0.0	0.6	1.4	2.0	3.8	0.08
**Dry fruit, nuts and seeds**	**1.8**	**3.4**	**0.0**	**0.2**	**0.3**	**1.7**	**8.8**	**0.09**
Dry fruit	0.4	1.1	0.0	0.0	0.1	0.1	2.4	0.02
Nuts and seeds	1.4	3.1	0.0	0.2	0.2	1.1	8.1	0.07
**Sweets products**	**117.9**	**123.4**	**3.5**	**35.6**	**84.2**	**156.6**	**336.8**	**5.68**
Sugar, non-chocolate confectionery	0.4	0.9	0.0	0.1	0.2	0.3	1.5	0.02
Chocolate, candy bars, etc.	2.2	3.5	0.0	0.0	0.8	2.5	8.8	0.11
Ice-cream	4.2	4.3	0.0	1.1	3.2	6.4	12.7	0.20
Cakes, pies and pastries	75.9	100.6	0.0	13.4	40.3	116.5	255.0	3.66
Biscuits, dry cakes	35.3	42.4	0.0	0.0	17.7	59.6	131.6	1.70
**Oils and fats**	**8.7**	**11.1**	**0.0**	**1.3**	**5.1**	**11.9**	**28.3**	**0.42**
Vegetables fats and oils (non-olive)	0.6	1.3	0.0	0.2	0.4	0.8	2.0	0.03
Olive oil	0.0	0.0	0.0	0.0	0.0	0.0	0.1	0.00
Butter and other animal fats	8.0	10.8	0.0	0.6	4.3	10.9	27.5	0.39
**Beverages**	**19.0**	**26.9**	**2.0**	**5.0**	**10.5**	**21.5**	**59.6**	**0.92**
Coffee and tea	3.6	3.1	0.6	1.8	2.8	4.4	8.9	0.17
Wine	1.1	1.7	0.0	0.0	0.3	1.8	3.8	0.05
Red wine	0.6	1.1	0.0	0.0	0.0	0.6	3.2	0.03
White wine	0.5	1.1	0.0	0.0	0.0	0.4	3.4	0.02
Aperitif wines and beers	3.3	12.5	0.0	0.0	0.6	2.2	14.2	0.16
Spirits and liqueurs	0.1	0.3	0.0	0.0	0.0	0.0	0.3	0.00
Fruit juices	5.3	13.9	0.0	0.0	0.8	6.1	22.4	0.26
Soft drinks	5.6	16.8	0.0	0.0	0.0	3.7	25.7	0.27

[1]Bold characters relate to main food categories

Supplemental Table S4. Distribution of potassium daily dietary intake in men (in mg/day) and percentage contribution (%) of each food to total intake.

**Food**[1]	**Mean**	**SD**	**P5**	**P25**	**P50**	**P75**	**P95**	**%**
**Total intake**	**3438.3**	**1047.4**	**2047.2**	**2706.0**	**3341.6**	**3909.2**	**5326.6**	**100**
**Cereals and cereal products**	**432.6**	**215.6**	**113.9**	**283.5**	**406.9**	**553.2**	**815.3**	**12.58**
Pasta and other grains	178.3	115.3	12.7	102.5	162.3	230.8	377.8	5.19
Rice	9.0	12.6	0.0	1.9	5.9	10.5	30.3	0.26
Bread and rolls	158.5	138.6	0.0	33.8	134.5	229.7	435.2	4.61
Pizza, crackers, and other salty snacks	86.8	67.7	7.9	38.5	80.3	110.2	192.4	2.52
**Meat and meat products**	**637.5**	**323.9**	**165.3**	**425.4**	**600.1**	**797.2**	**1295.0**	**18.54**
Red meat	326.4	210.5	54.0	178.4	290.5	432.2	772.6	9.49
White meat	163.7	136.2	5.8	68.0	135.5	230.4	423.4	4.76
Processed meat	139.7	117.6	14.3	58.4	106.9	187.4	374.5	4.06
Offal	7.8	18.9	0.0	0.0	0.0	7.2	29.0	0.23
**Milk and dairy products**	**334.9**	**353.5**	**18.0**	**76.3**	**251.1**	**469.6**	**908.7**	**9.74**
Milk and yogurt	290.1	350.7	0.0	19.2	206.4	417.7	846.2	8.44
Cheese	44.7	37.7	5.2	18.5	35.6	59.1	112.0	1.30
Fresh cheese	14.8	19.2	0.0	1.5	9.4	20.0	50.3	0.43
Aged cheese	30.0	27.1	3.1	11.4	22.8	41.4	78.2	0.87
**Eggs**	**17.4**	**13.4**	**1.6**	**8.3**	**14.8**	**23.9**	**42.5**	**0.51**
**Fish and seafood**	**108.9**	**82.7**	**10.4**	**49.7**	**87.9**	**141.2**	**280.8**	**3.17**
Fish	97.6	77.5	9.3	42.4	80.4	128.2	274.2	2.84
Preserved and tinned fish	34.7	32.7	0.0	11.4	28.1	43.4	94.3	1.01
Non-piscivorous fish	38.6	47.5	0.0	5.4	22.3	52.0	156.0	1.12
Piscivorous fish	24.3	34.3	0.0	1.1	11.8	38.3	83.3	0.71
Crustaceans and mollusks	11.2	15.3	0.0	1.1	5.1	18.2	36.8	0.33
**All vegetables**	**513.1**	**291.2**	**135.4**	**331.2**	**454.4**	**641.3**	**1111.5**	**14.92**
Leafy vegetables	95.8	77.8	8.8	40.4	74.0	127.3	264.8	2.79
Tomatoes	264.8	199.5	35.9	132.1	217.4	357.2	632.6	7.70
Root vegetables	80.6	82.4	8.8	29.0	55.2	101.8	239.5	2.34
Cabbage	12.1	25.3	0.0	0.0	4.1	13.2	55.0	0.35
Other vegetables	59.8	44.9	8.5	26.9	46.3	84.5	150.7	1.74
**Mushrooms**	**12.8**	**21.3**	**0.0**	**1.0**	**6.1**	**20.4**	**43.8**	**0.37**
**Pulses**	**196.1**	**194.4**	**7.0**	**62.4**	**142.9**	**258.6**	**580.6**	**5.70**
**Potatoes**	**103.5**	**107.1**	**6.9**	**43.4**	**73.4**	**124.9**	**311.0**	**3.01**
**Fresh fruit**	**512.1**	**309.4**	**81.1**	**313.2**	**463.8**	**667.0**	**1105.1**	**14.89**
Citrus fruit	403.7	259.1	68.6	227.2	358.2	523.2	930.7	11.74
All other fruit	108.4	84.8	0.0	46.4	97.1	143.7	260.0	3.15
**Dry fruit, nuts and seeds**	**13.2**	**21.5**	**0.0**	**1.4**	**2.2**	**13.1**	**60.7**	**0.38**
Dry fruit	3.8	10.2	0.0	0.0	0.8	0.8	23.4	0.11
Nuts and seeds	9.4	15.7	0.0	1.4	1.4	9.7	49.3	0.27
**Sweets products**	**161.3**	**147.1**	**5.3**	**59.5**	**127.3**	**207.5**	**433.6**	**4.69**
Sugar, non-chocolate confectionery	25.9	27.0	0.0	5.9	18.3	41.4	73.9	0.75
Chocolate, candy bars, etc.	36.7	63.8	0.0	0.0	9.8	42.9	169.4	1.07
Ice-cream	20.2	25.3	0.0	3.7	11.8	29.5	71.9	0.59
Cakes, pies and pastries	51.5	93.3	0.0	0.0	19.1	71.4	176.9	1.50
Biscuits, dry cakes	27.0	35.3	0.0	0.0	10.5	49.3	98.6	0.79
**Oils and fats**	**1.3**	**1.2**	**0.1**	**0.4**	**0.9**	**1.8**	**3.5**	**0.04**
Vegetables fats and oils (non-olive)	0.1	0.1	0.0	0.0	0.0	0.1	0.3	0.00
Olive oil	0.1	0.1	0.0	0.1	0.1	0.2	0.3	0.00
Butter and other animal fats	1.1	1.2	0.0	0.2	0.7	1.6	3.3	0.03
**Beverages**	**393.7**	**263.7**	**67.0**	**225.3**	**341.9**	**510.5**	**844.1**	**11.45**
Coffee and tea	160.6	138.6	0.0	71.7	147.0	223.0	363.0	4.67
Wine	112.4	113.4	0.0	8.1	84.2	177.0	336.9	3.27
Red wine	82.6	102.1	0.0	0.7	39.4	157.4	265.6	2.40
White wine	29.9	49.4	0.0	0.0	9.1	44.4	124.8	0.87
Aperitif wines and beers	33.0	70.5	0.0	0.5	4.2	31.8	159.1	0.96
Spirits and liqueurs	0.3	0.9	0.0	0.0	0.0	0.1	2.4	0.01
Fruit juices	79.6	152.6	0.0	0.0	17.6	88.0	376.2	2.32
Soft drinks	7.9	17.7	0.0	0.0	0.0	10.0	35.1	0.23

[1]Bold characters relate to main food categories

Supplemental Table S5. Distribution of potassium daily dietary intake in women (in mg/day) and percentage contribution (%) of each food to total intake.

**Food**[1]	**Mean**	**SD**	**P5**	**P25**	**P50**	**P75**	**P95**	**%**
Total intake	***3310.7***	***1186.4***	***1742.8***	***2514.1***	***3082.8***	***3836.6***	***5782.4***	***100***
**Cereals and cereal products**	**357.1**	**186.5**	**105.2**	**226.8**	**340.6**	**455.6**	**631.1**	**10.79**
Pasta and other grains	119.0	84.5	8.1	61.4	104.3	164.0	278.0	3.59
Rice	7.2	9.6	0.0	1.5	4.7	7.9	25.6	0.22
Bread and rolls	139.5	133.3	0.0	40.7	108.0	195.0	377.0	4.21
Pizza, crackers, and other salty snacks	91.3	62.4	11.4	47.4	82.3	121.2	211.3	2.76
**Meat and meat products**	**525.0**	**302.0**	**127.1**	**326.6**	**468.4**	**669.3**	**1090.1**	**15.86**
Red meat	255.1	169.9	26.9	127.7	225.8	353.9	567.0	7.71
White meat	146.2	138.0	0.0	54.8	103.8	203.7	417.4	4.42
Processed meat	118.0	98.4	9.7	51.3	94.4	155.4	296.1	3.56
Offal	5.7	14.6	0.0	0.0	0.0	3.6	29.0	0.17
**Milk and dairy products**	**437.3**	**445.0**	**29.4**	**202.4**	**342.3**	**536.2**	**1094.3**	**13.21**
Milk and yogurt	394.7	435.0	0.0	165.0	292.2	484.2	1040.6	11.92
Cheese	42.7	36.4	3.4	19.5	34.4	53.7	105.5	1.29
Fresh cheese	20.8	26.2	0.0	5.5	13.4	26.5	60.1	0.63
Aged cheese	21.8	19.7	0.3	8.8	17.1	29.3	54.8	0.66
**Eggs**	**18.4**	**13.7**	**1.9**	**9.1**	**16.7**	**25.5**	**37.3**	**0.56**
**Fish and seafood**	**106.0**	**88.9**	**13.8**	**49.8**	**86.6**	**133.8**	**265.4**	**3.20**
Fish	94.1	82.0	9.3	41.2	76.7	120.2	234.6	2.84
Preserved and tinned fish	29.0	41.0	0.0	9.3	20.0	40.0	82.6	0.88
Non-piscivorous fish	36.9	43.0	0.0	6.0	25.6	53.9	111.0	1.11
Piscivorous fish	28.2	40.2	0.0	1.9	15.3	41.0	96.3	0.85
Crustaceans and mollusks	11.9	16.8	0.0	1.1	6.0	16.5	52.4	0.36
**All vegetables**	**501.8**	**291.5**	**145.7**	**295.5**	**453.0**	**632.8**	**1134.3**	**15.16**
Leafy vegetables	108.3	91.7	10.5	41.9	80.7	153.5	289.8	3.27
Tomatoes	209.7	159.6	17.3	87.8	173.8	294.1	521.8	6.33
Root vegetables	92.4	94.3	10.6	32.6	64.8	111.6	303.3	2.79
Cabbage	13.9	19.5	0.0	1.3	6.8	20.4	56.2	0.42
Other vegetables	77.4	57.6	13.1	34.3	63.1	105.8	197.9	2.34
**Mushrooms**	**13.0**	**19.4**	**0.0**	**1.5**	**5.1**	**20.4**	**43.8**	**0.39**
**Pulses**	**182.5**	**181.8**	**5.0**	**64.4**	**135.8**	**241.0**	**513.7**	**5.51**
**Potatoes**	**96.2**	**90.5**	**9.9**	**41.0**	**72.6**	**131.0**	**256.7**	**2.91**
**Fresh fruit**	**544.2**	**318.9**	**102.1**	**315.6**	**511.0**	**718.3**	**1150.4**	**16.44**
Citrus fruit	436.3	268.0	80.6	250.0	401.6	574.5	909.2	13.18
All other fruit	107.8	86.5	2.4	44.4	99.3	139.3	260.0	3.26
**Dry fruit, nuts and seeds**	**11.4**	**21.6**	**0.0**	**1.4**	**2.2**	**10.5**	**58.6**	**0.34**
Dry fruit	2.8	8.3	0.0	0.0	0.8	0.8	17.1	0.08
Nuts and seeds	8.6	19.1	0.0	1.4	1.4	6.9	49.3	0.26
**Sweets products**	**175.8**	**147.7**	**25.6**	**81.0**	**136.4**	**227.8**	**423.6**	**5.31**
Sugar, non-chocolate confectionery	29.0	37.8	0.0	6.4	18.7	41.4	83.9	0.88
Chocolate, candy bars, etc.	41.9	67.4	0.0	0.0	15.1	48.9	169.4	1.27
Ice-cream	21.2	21.6	0.0	5.7	16.0	31.9	64.0	0.64
Cakes, pies and pastries	54.6	72.3	0.0	9.6	29.0	83.7	183.3	1.65
Biscuits, dry cakes	29.2	35.1	0.0	0.0	14.7	49.3	108.9	0.88
**Oils and fats**	**1.0**	**1.0**	**0.1**	**0.3**	**0.7**	**1.3**	**2.8**	**0.03**
Vegetables fats and oils (non-olive)	0.0	0.1	0.0	0.0	0.0	0.1	0.1	0.00
Olive oil	0.1	0.1	0.0	0.1	0.1	0.2	0.3	0.00
Butter and other animal fats	0.8	1.0	0.0	0.1	0.5	1.2	2.5	0.02
**Beverages**	**341.1**	**281.7**	**70.0**	**176.8**	**271.0**	**420.0**	**775.4**	**10.30**
Coffee and tea	196.9	177.3	9.5	95.1	171.9	249.9	445.4	5.95
Wine	42.2	67.2	0.0	0.0	8.6	66.2	169.5	1.27
Red wine	29.0	56.7	0.0	0.0	1.5	30.6	157.4	0.88
White wine	13.2	30.2	0.0	0.0	0.4	9.9	88.7	0.40
Aperitif wines and beers	22.7	84.5	0.0	0.0	3.7	14.8	96.2	0.69
Spirits and liqueurs	0.1	0.3	0.0	0.0	0.0	0.0	0.3	0.00
Fruit juices	71.6	154.6	0.0	0.0	14.6	80.4	263.7	2.16
Soft drinks	7.7	22.9	0.0	0.0	0.0	5.0	35.1	0.23

[1]Bold characters relate to main food categories
